# Understanding copy number variations through their genes: a molecular view on 16p11.2 deletion and duplication syndromes

**DOI:** 10.3389/fphar.2024.1407865

**Published:** 2024-06-14

**Authors:** Roberta Leone, Cecilia Zuglian, Riccardo Brambilla, Ilaria Morella

**Affiliations:** ^1^ Università di Pavia, Dipartimento di Biologia e Biotecnologie “Lazzaro Spallanzani”, Pavia, Italy; ^2^ Cardiff University, School of Biosciences, Neuroscience and Mental Health Innovation Institute, Cardiff, United Kingdom

**Keywords:** 16p11.2 CNV, basal ganglia, MAPK3, neurodevelopmental disorders, animal models, iPSC (induced pluripotent stem cell), excitation/inhibition (E/I) imbalance, intellectual disability

## Abstract

Neurodevelopmental disorders (NDDs) include a broad spectrum of pathological conditions that affect >4% of children worldwide, share common features and present a variegated genetic origin. They include clinically defined diseases, such as autism spectrum disorders (ASD), attention-deficit/hyperactivity disorder (ADHD), motor disorders such as Tics and Tourette’s syndromes, but also much more heterogeneous conditions like intellectual disability (ID) and epilepsy. Schizophrenia (SCZ) has also recently been proposed to belong to NDDs. Relatively common causes of NDDs are copy number variations (CNVs), characterised by the gain or the loss of a portion of a chromosome. In this review, we focus on deletions and duplications at the 16p11.2 chromosomal region, associated with NDDs, ID, ASD but also epilepsy and SCZ. Some of the core phenotypes presented by human carriers could be recapitulated in animal and cellular models, which also highlighted prominent neurophysiological and signalling alterations underpinning 16p11.2 CNVs-associated phenotypes. In this review, we also provide an overview of the genes within the 16p11.2 locus, including those with partially known or unknown function as well as non-coding RNAs. A particularly interesting interplay was observed between MVP and MAPK3 in modulating some of the pathological phenotypes associated with the 16p11.2 deletion. Elucidating their role in intracellular signalling and their functional links will be a key step to devise novel therapeutic strategies for 16p11.2 CNVs-related syndromes.

## Introduction

Neurodevelopmental disorders (NDDs) include conditions with a wide range of neuropsychiatric symptoms that are now believed to originate from alterations at the cortical and subcortical level during both prenatal and early postnatal brain development. Symptoms are highly variable, especially in the predominant idiopathic forms, but the core components are normally associated to intellectual disability (ID), autism spectrum disorder (ASD), attention-deficit/hyperactivity disorder (ADHD) and epilepsy. Associated psychiatric symptoms may include depression and anxiety, speech delays and in the most severe cases, schizophrenia and/or bipolar disorder. In syndromic forms, non-psychiatric symptoms may also occur, including metabolic dysfunctions, alterations in brain size, and cardio-facial-cutaneous malformations (Mitchell, 2011).

We know from previous literature (revised in (Backhausen et al., 2022) that cortical and subcortical grey matter formation follows a structured developmental pattern, presenting an initial increase in childhood that is followed by a decrease during adolescence. This developmental trajectory supports the mechanisms of reinforcement of important connection through learning and the elimination of redundant synapses during maturation, and it is differently affected in NDDs. For instance, while ADHD is characterised by a general reduction in the volume and surface area of basal ganglia and prefrontal cortex (PFC) (Shaw et al., 2014; Hoogman et al., 2017), children with ID present a general decrease in brain size throughout childhood and adolescence, but prefrontal and cingulate areas present higher volumes than healthy peers (Ma et al., 2021). A generalized increase in frontal cortical volumes has also been described in ASD patients within the first 2 years of age (Courchesne et al., 2011): a tendency that reverts in adulthood, when ASD brains show a higher rate of structural decline (Wallace and Rogers, 2010; Courchesne et al., 2011; Lange et al., 2015), although maintaining an abnormal growth rate in the basal ganglia (Langen et al., 2009; Wegiel et al., 2014).

Interestingly, in Schizophrenia (SCZ), a reduction in cortical volume is observed, mostly in the frontal, prefrontal and temporal lobes, accompanied by a reduction in the volume of basal ganglia and a more peculiar enlargement of the ventricles (Shenton et al., 2001; Fornito et al., 2009; Kuo and Pogue-Geile, 2019; Cai et al., 2022). While the onset of the symptoms is delayed in SCZ compared to other NDDs, morphological abnormalities have been described prior to symptoms appearance and become more severe over time, thus supporting the neurodevelopmental origin of this disease (Rund, 2009; Owen et al., 2011).

Although brain development differently deviates from its physiological developmental trajectory in different NDDs, affected areas recurrently belong to the thalamus-striatal-prefrontal axis (Di Martino et al., 2011; Prat et al., 2016; Roy et al., 2021). Morphological brain abnormalities constitute an endophenotype common to all NDDs, accompanied by a strong comorbidity of other symptoms, including cognitive, motor and social impairments (Eberhard et al., 2022), as well as seizures (Chow et al., 2019; Watkins et al., 2022) and sex biases (Santos et al., 2022). An endophenotypes can be caused by different genetic variations which affect one or more neural circuits, independently leading to an overall effect that is common to multiple clinical entities (Cannon and Keller, 2006). Results from genome-wide association studies described a strong correlation between NDDs and single genomic variations in functionally related sets of genes involved in neurodevelopmental processes, synaptic plasticity, learning and memory (Cross-Disorder Group of the Psychiatric Genomics, 2013; Ripke et al., 2013; Doherty and Owen, 2014; Fromer et al., 2014). These shared risk factors are responsible not only for symptoms comorbidity among patients with different diagnoses of NDDs, but they also increase the risk of developing NDDs in families where the same or a different NDD is already present (Larsson et al., 2005; Daniels et al., 2008; Sullivan et al., 2012; Larsson et al., 2013). However, it is very difficult to ascribe all the clinical manifestations of different NDDs to single gene variants, although several cases of direct causality do exist, such as FMR1 as a genetic cause of ASD (Fyke and Velinov, 2021), NRG1 and DISC1 for SCZ (Dahoun et al., 2017; Zhang et al., 2017) and FOXP2 in ADHD (Faraone and Larsson, 2019). Most likely, single genes found associated with NDDs should play major roles in one or more biological processes critical for brain development and function, including connectivity, synaptic transmission, and neuronal signalling.

Genetic causes of NDDs also include copy number variations (CNVs), rare genetic variants in which either deletion or duplication (less often triplication) of an entire chromosomal portion may occur (for a general review, see (Grayton et al., 2012). These chromosomal rearrangements may originate from genetic transmission to offspring or may be *de-novo* mutations and constitute a significant burden in the onset of NDDs (Sebat et al., 2007; Walsh et al., 2008; Sonderby et al., 2022). They include several genes of both known and unknown function, potentially interfering with multiple, interlinked, molecular pathways. Although CNVs are less common than single gene variations, they constitute a much stronger risk factor in the development of NDDs, with a penetrance from 10% to 100% in some cases (Kirov, 2015). Interestingly, penetrance of symptoms can be highly variable within the same CNV carrier population, with subjects with no notable phenotypes while others severely affected. Gene dosage does not usually help in understanding symptom severity, since opposite variations on the same genomic region often lead to similar phenotypes (Niarchou et al., 2019; Zarrei et al., 2019).

This genomic complexity represents a formidable challenge to understand the relative contribution of each gene, and its likely interactions with the nearby CNV genes. However, it strongly limits our understanding of the pathological mechanisms as well as the devising of effective therapies. Furthermore, non-coding RNAs that are commonly found in CNVs may also be relevant for the onset of NDD phenotypes.

In this review, we will focus our attention on the 16p11.2 CNV (Rein and Yan, 2020). Deletions (DEL) and duplications (DUP) at the human 16p11.2 breakpoints (BP) four to five chromosomal region account for leading causes of neurodevelopmental disorders and intellectual disabilities worldwide, with an estimated of three in 10,000 people for each syndrome. Individuals with deletions or duplications are diagnosed with intellectual disabilities and psychiatric disorders with a likelihood of 50% and 60%, respectively (Niarchou et al., 2019).

CNVs on 16p11.2 are most frequently associated with ASD and other NDDs than do other CNVs. Data pooled from different studies found a frequency of 0.35%, 0.21%, 0.17%, and 0.17% for 16p11.2 deletions, 16p11.2 duplications, 1q21.1 duplications, and 15q11.2–13.1 duplications, respectively, in the onset of ASD, ID, ADHD (Mollon et al., 2023). It is noteworthy that deletions on the chromosomal region 22q11.2 also constitute a strong risk factor for SCZ and, to a less extent, ASD (Bassett et al., 2003). Interestingly, some genes on 16p11.2 and 22q11.2 regions belong to common molecular pathways. For instance, ERK1 is located on 16p11.2, ERK2 on 22q11.2, and the reciprocal differential expression level for these two effectors of the MAPK signalling cascade has previously been associated with cognitive impairments and neurodevelopmental deficits (Mazzucchelli et al., 2002; Pucilowska et al., 2018; Indrigo et al., 2023). Other examples include the presence of different members of the T-Box family (TBX6 in 16p11.2, TBX1 in 22q11.2), implicated in embryogenesis and development, and members of the H3.3 histone chaperone complex HIRA (HIRA on 22q11.2, HIRIP3 on 16p11.2) responsible for chromatin remodelling and gene transcription.

Behavioural phenotypes, often observed in both DEL and DUP carriers, are in the domain of speech, intellectual disability, and autistic traits, with DUP carriers bearing greater cognitive impairments in full-scale IQ, verbal IQ, and performance IQ compared with DEL carriers (Chawner et al., 2021). Importantly, both DEL and DUP carriers have a significant risk of developing seizures, suggesting that an altered brain development may lead to changes in the excitation/inhibition balance. In addition, DUP carriers may be susceptible to psychosis and bipolar disorder that are generally absent in DEL carriers (Rein and Yan, 2020).

Here, we describe the pathological phenotypes, and we review the recent literature on cellular and animal models of 16p11.2 CNVs. We subsequently hypothesize a link between the genes in the 16p11.2 region and their effect on specific molecular pathways. We also suggest potential interventions to rescue the deficits affecting 16p11.2 CNVs carriers.

## Clinical profile of 16p11.2 CNVs

CNVs on 16p11.2 chromosomic region were first correlated to autism spectrum disorder after a large study on a patients’ database in 2008, with a frequency of three in 10000 (Weiss et al., 2008). Deletions (DEL) occur *de novo* in most cases (71%), while duplications (DUP) are mainly familial (D'Angelo et al., 2016). Variations in the 16p11.2 locus lead to heterogeneous clinical effects, including intellectual disability (ID), autism (ASD), attention deficit hyperactivity disorder (ADHD), epilepsy, language and motor delays (Weiss et al., 2008; Shinawi et al., 2010; Hanson et al., 2015; D'Angelo et al., 2016; Green Snyder et al., 2016; Steinman et al., 2016; Niarchou et al., 2019; Rein and Yan, 2020), which appear in different proportions between DEL and DUP patients (reviewed in (Oliva-Teles et al., 2020)). Moreover, duplications constitute an additional risk factor for schizophrenia (SCZ) (McCarthy et al., 2009; Shinawi et al., 2010; Niarchou et al., 2019; Zarrei et al., 2019; Rein and Yan, 2020).

In the context of 16p11.2 CNVs, ASD and SCZ are often considered as opposite conditions of dosage-dependent modifications of gene expression (Crespi et al., 2010). In addition, cognitive studies showed that the 16p11.2 deletion is strongly associated with impaired verbal IQ, deficits in verbal letter and category fluency tests, consistently to autism symptomatology, while duplication affects spatial working memory and executive functions (Stefansson et al., 2014), as observed in schizophrenic patients (Park and Holzman, 1992). In other cases, 16p11.2 deletion and duplication similarly affect cognition, but with different severity degrees. For instance, duplications are usually characterised by higher variance, suggesting the possible contribution of additional familial factors (D'Angelo et al., 2016; Niarchou et al., 2019). The body mass index (BMI) and the brain size are also differently affected in DEL and DUP carriers, and negatively correlate with gene dosage (McCarthy et al., 2009; Jacquemont et al., 2011; Zufferey et al., 2012; Qureshi et al., 2014; Martin-Brevet et al., 2018), as well as facial dysmorphisms (Shinawi et al., 2010). From a neuroanatomical point of view, patients affected by 16p11.2 CNVs display structural abnormalities similar to those described in NDDs. More specifically, magnetic resonance imaging (MRI) and diffusion tensor imaging (DTI) studies on DEL children reported increased white matter volume and fibre density, with reduced orientation dispersion in the callosum and internal/external capsules, compared to healthy controls (Owen et al., 2014). The opposite effect was observed in DUP carriers (Chang et al., 2016). Altogether, these observations are consistent with the ASD phenotype and other NDDs (Owen et al., 2014; Chang et al., 2016). Importantly, the observed changes in fibre density affect the development and function of connections between brain areas involved in language, locomotion, and socio-emotional behaviours (Maillard et al., 2024).

However, 16p11.2 DEL and DUP carriers also share common pathological phenotypes, such as epilepsy. This is typically observed during the first year of life, easily responds to antiepileptic medications, and usually decreases in severity or disappears during childhood (Shinawi et al., 2010).

## Animal models of 16p11.2 deletion and duplication

**FIGURE 1:**
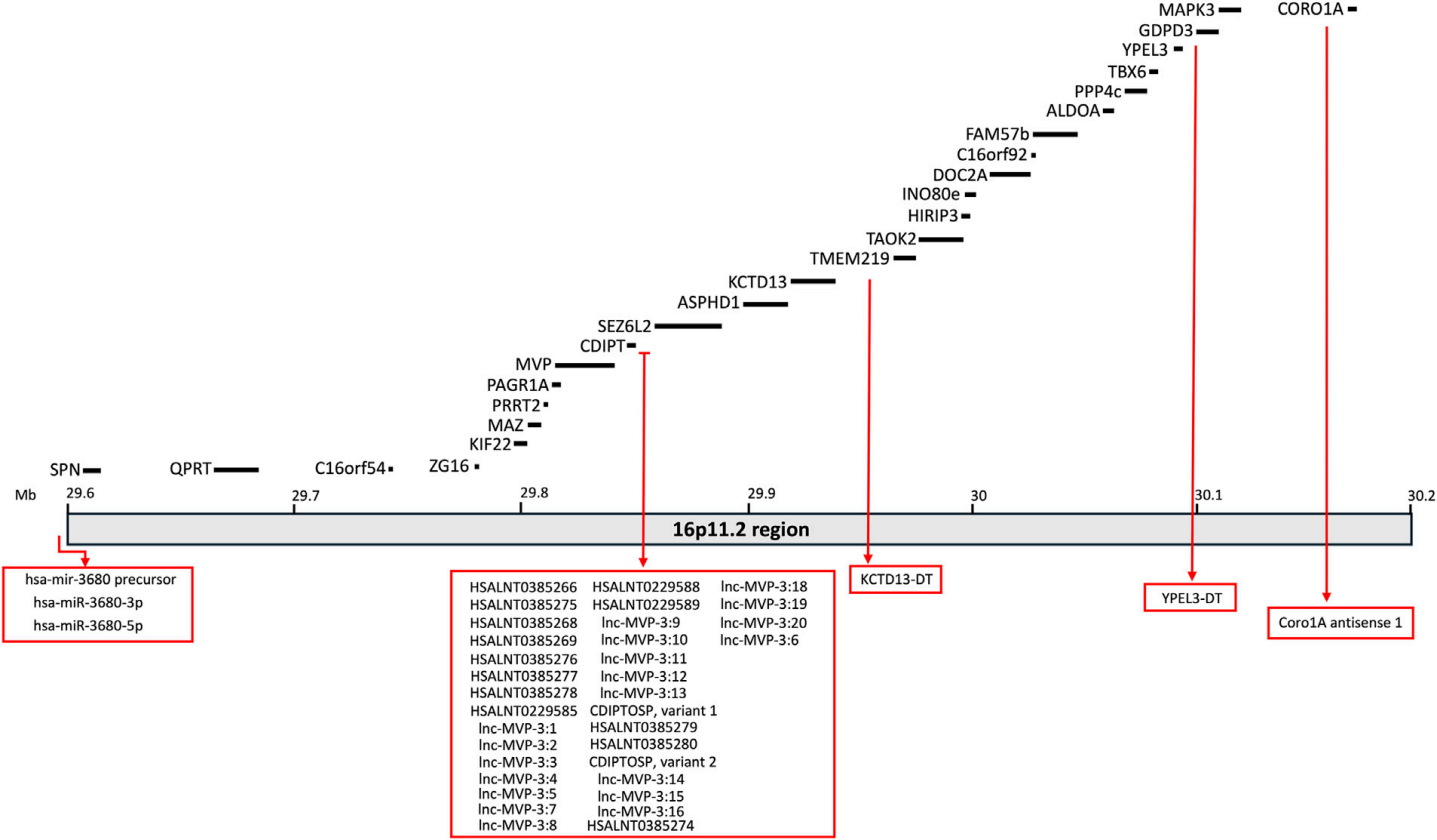
Schematic representation of the 16p11.2 locus containing 27 protein-coding genes. Non-coding RNAs mapping on this region are also listed in the rex boxes.

The clinical profile associated with 16p11.2 CNVs is very heterogeneous and suggests the presence of multifactorial effects in determining the pathological phenotypes of 16p11.2 DEL and DUP carriers. The systematic evaluation of animal models carrying the 16p11.2 CNVs has allowed a deeper investigation of the cellular and molecular pathways involved in the pathophysiology of 16p11.2 CNVs, as well as the specific functions of the genes within the 16p11.2 locus. In the human genome, the 16p11.2 locus is a region of approximately 600kb, defined by breakpoints 4 and 5 (BP4-BP5), which is conserved on mouse syntenic region located on chromosome 7F3 (Rein and Yan, 2020). The BP4-BP5 common rearrangements encompass 27 unique protein coding-genes (see Table 1; Figure 1) and multiple copies of BOLA2/2B, SLX1A/1B, SULT1A3/4 and NPIP.

**TABLE 1:** List of genes within the 16p11.2 chromosomal region, relative functions, and involvement in the 16p11.2 CNVs.

Gene	Full name	OMIM ID	Function	Involvement in 16p11.2 CNVs	Refs
SPN	Sialophorin	182160	T-cells activation in immune function	Overexpressed in regions with decreased fiber density in male 16p11.2 DEL mice	Kumar et al., 2018 (PMID: 29844452)
				Correlates with reduced lymphocytes count in 16p11.2 DEL patients presenting a concomitant low dosage of BOLA2 duplicone	Giannuzzi et al., 2022 (PMID: 35715439); Giannuzzi et al., 2019 (PMID: 31668704)
QPRT	Quinolinate phosphoribosyltransferase	606248	Catabolism of quinolinate during NAD synthesis	Altered QPTR gene dosage influences neuronal differentiation and excitatory/inhibitory network development	Haslinger et al., 2018 (PMID: 30443311)
C16orf54	Chromosome 16 open reading frame 54	Not available	Unknown, found dysregulated in various tumours	Unknown	Du et al., 2022 (PMID: 36118669); Ding et al., 2023 (PMID: 37766321)
ZG16	Zymogen granule protein 16	617311	Putative immune checkpoint inhibitor in cancer	ZG16 deletion in mice is associated with increased size of different brain areas	Kretz et al., 2023 (PMID: 37968726); Meng et al., 2022 (PMID: 35831911)
KIF22	Kinesin family member 22	603213	Regulator of mitotic spindle, microtubule stability and CDC25C expression	Involved in movement defects and deficient axon development in Zebrafish	Blaker-Lee et al., 2012 (PMID: 22566537)
				Required for synaptic wiring in *Drosophila* neuromuscular junction	Park et al., 2016 (PMID: 26924931)
				Highly expressed in progenitors, potential involvement in neurogenesis	Morson et al., 2021 (PMID: 33825894)
MAZ	MYC-associated zinc finger protein	600999	Transcription factor, involved in gene expression and signal transduction	Involved in movement defects in Zebrafish	Blaker-Lee et al., 2012 (PMID: 22566537)
				Regulates the differentiation of neuronal/glial profiles during CNS development via interaction with various signalling pathways	Ortabozkoyun et al., 2022 (PMID: 35145304); Haller et al., 2018 (PMID: 29432158); Medina Martinez et al., 2020 (PMID: 32571845); Morson et al., 2021 (PMID: 33825894)
PRRT2	Proline Rich Transmembrane Protein 2	614386	Synapse formation during development, regulation of presynaptic Ca2+ influx and neurotransmitter release	Enriched in Drd2+ MSNs in 16p11.2 DEL mice	Portmann et al., 2014 (PMID: 24794428)
				PRRT2 mutations are associated with benign familial infantile seizures and autistic developmental regression by 15 months of age	Zhang et al., 2024 (PMID: 38406554)
PAGR1a	Pax-interacting protein 1-associated glutamate rich protein 1a	612033	Component of the histone methyltransferase MLL2/MLL3 complex, possible role in DNA damage response	Correlation with ataxia and seizures in 16p11.2 DEL patients	Padmanabha et al., 2024 (PMID: 38091792); Vlaskamp et al., 2019 (PMID: 30125676)
				Correction of PRRT2 copy number in 16p11.2 DUP mice corrects neural circuits defects, seizure susceptibility and social deficits	Forrest et al., 2023 (PMID: 36808153)
				Highly expressed in neural progenitors	Morson et al., 2021 (PMID: 33825894)
				Homozygous missense mutation in PAGR1a gene is associated with a severe neurodevelopmental phenotype	Daum et al., 2022 (PMID: 34585832)
				Mice lacking one copy of Pagr1a show abnormal development of extraembryonic tissues	Kumar et al., 2014 (PMID: 24633704)
				In Zebrafish, loss of function of Pagr1a is associated with reduced brain ventricle size and less defined midbrain-hindbrain boundary	Blaker-Lee et al., 2012 (PMID: 22566537)
MVP	Major vault protein	605088	Main component of the vault organelle; potential scaffold for ERK and PI3K/AKT/mTOR signalling	MVP loss of function is associated with abnormal body length and defective neural tube in Zebrafish	Blaker-Lee et al., 2012 (PMID: 22566537)
				MVP is overexpressed in regions with increased functional anisotropy in males 16p11.2 DEL mice	Kumar et al., 2018 (PMID: 29844452)
				MVP is responsible for sex-specific structural changes in the striatum of male 16p11.2 DEL mice	Kim et al., 2024 (PMID: 38278994)
				MVP is a main driver of brain neuroanatomical phenotypes	Kretz et al., 2023 (PMID: 37968726)
CDIPT	CDP-diacylglycerol–inositol 3-phosphatidyltransferase	605893	Catalyzes the biosynthesis of phosphatidylinositol	CDIPT knock-down in *Drosophila* leads to altered development of the neuromuscular junction	Iyer et al., 2018 (PMID: 29959322)
				A missense mutation in CDIPT gene in Zebrafish is associated with cataract and lack of cone photoreceptors	Murphy et al., 2011 (PMID: 21722635)
SEZ6L2	Seizure-related 6 homolog like 2	616667	Transmembrane protein required by Cathepsin D for its endosome/lysosome localization; regulates neurite outgrowth	Enriched in Drd2+ MSNs in 16p11.2 DEL mice	Portmann et al., 2014 (PMID: 24794428)
				Overexpressed in regions with increased functional anisotropy in male 16p11.2 DEL mice	Kumar et al., 2018 (PMID: 29844452); Kim et al., 2024 (PMID: 38278994)
ASPHD1	Aspartate beta-hydroxylase domain containing 1	Not available	Unknown	Unknown	Not available
KCTD13	Potassium channel tetramerization domain containing 13	608947	Forms a complex with Cul3 ubiquitin E3 ligase to target RhoA for ubiquitination and degradation	Loss of function of KCTD13 causes deficient axon tracts in Zebrafish	Blaker-Lee et al., 2012 (PMID: 22566537)
				KCTD13 suppression induces macrocephaly, while its overexpression induces microcephaly in Zebrafish, in epistasis with MAPK3 and MVP	Golzio et al., 2012 (PMID: 22596160)
				KCTD13 interacts with ciliopathy-associated genes in Zebrafish	Migliavacca et al., 2015 (PMID: 25937446)
				KCTD13 KO mice show a reduction of dendritic lenght, complexity and spine density due to increased RhoA levels	Escamilla et al., 2017 (PMID: 29088697)
				KCTD13 knockdown causes seizure phenotypes decreased complexity in dendritic arborization. KCTD13 KO *Drosophila* show aberrant axonal-sympathetic targeting	Iyer et al., 2018 (PMID: 29959322)
				Enriched in Drd2+ MSNs in 16p11.2 DEL mice	Portmann et al., 2014 (PMID: 24794428)
TMEM219	Transmembrane protein 219	620290	Mediates apoptosis and tumour suppression in prostate and breast cancer. Inhibits oxidant-induced apoptosis in the lung and induces TGF-β1 by interacting with chitinase-3-like-1	Unknown	Ingermann et al., 2010 (PMID: 20353938); Lee et al., 2016 (PMID: 27629921)
TAOK2	TAO kinase 2	613199	Role in dendritic arborization and synapse maturation	TAOK2 knock-down is associated with increased complexity of dendritic arbor in *Drosophila*	Iyer et al., 2018 (PMID: 29959322)
				TAOK2 is overexpressed in regions with increased functional anisotropy in male 16p11.2 DEL mice	Kumar et al., 2018 (PMID: 29844452)
				TAOK2 is responsible for sex-specific structural changes in the striatum of male 16p11.2 DEL mice	Kim et al., 2024 (PMID: 38278994)
				TAOK2 heterozygous deletion in mice causes a reduction in RhoA activity leading to neurodevelopmental and behavioural deficits	Richter et al., 2019 (PMID: 29467497)
				Ectopic expression of TAOK2α in 16p11.2 DEL mice rescues migration deficits of cortical neurons	Scharrenberg et al., 2022 (PMID: 36123424)
HIRIP3	HIRA interacting protein 3	603365	Unknown, part of the histone H3.3 chaperone complex with HIRA	Loss of function of HIRIP3 produces movement defects in Zebrafish	Blaker-Lee et al., 2012 (PMID: 22566537)
INO80e	Ino80 complex subunit E	Not available	Unknown, potential involvement in chromatin remodelling and DNA replication	Loss of function of Ino80e in Zebrafish is linked to abnormal body length and defective neural tube	Blaker-Lee et al., 2012 (PMID: 22566537)
				Specifically overexpressed in regions with decreased fiber density in male 16p11.2 DEL mice	Kumar et al., 2018 (PMID: 29844452)
DOC2A	Double C2-like domain-containing protein, alpha	604567	Ca^2+^ sensor involved in neurotransmitter release, mainly expressed in glutamatergic neurons	DOC2A-KO mice show abnormal morphology in the dentate gyrus neurons, defective neural activity in the hippocampus, repetitive behaviours and social deficits	Wang et al., 2023 (PMID: 37354460)
				Specifically overexpressed in regions with decreased fiber density in male DEL mice	Kumar et al., 2018 (PMID: 29844452)
C16orf92	Chromosome 16 open reading frame 92	618911	Testis-specific protein required for oocyte-sperm fusion	Unknown	Fujihara et al., 2020 (PMID: 32395885)
FAM57b	Family with sequence similarity 57, member B	615175	Mediates the production of lactosylceramide	Fam57b loss of function in Zebrafish causes movement defects in, no response to touch and deficient axon tracts	Blaker-Lee et al., 2012 (PMID: 22566537)
				Fam57b knock-down in *Drosophila* causes abnormal growth development of the neuromuscular junction	Iyer et al., 2018 (PMID: 29959322)
				Enriched in Drd2+ MSNs in 16p11.2 DEL mice	Portmann et al., 2014 (PMID: 24794428)
ALDOA	Aldolase A, fructose-bisphosphate	103850	Glycolitic enzyme catalyzing the conversion of fructose-1,6-bisphosphate to glyceraldehyde 3-phosphate and dihydroxyacetone phosphate	ALDOA loss of function in Zebrafish is associated with no response to touch	Blaker-Lee et al., 2012 (PMID: 22566537)
				ALDOA knock-down in *Drosophila* is associated with a reduction in climbing ability, defects in cell counts and patterning of different cell types	Iyer et al., 2018 (PMID: 29959322)
				Highly expressed in neural progenitors	Morson et al., 2021 (PMID: 33825894)
PPP4C	Protein phosphatase 4, catalytic subunit	602035	Regulation of microtubule growth, DNA damage checkpoint recovery, apoptosis and TNF-alpha signalling	PPP4C loss of function causes movement defects in Zebrafish and deficient axon tracts	Blaker-Lee et al., 2012 (PMID: 22566537)
				PPP4C is specifically overexpressed in regions with decreased fiber density in male 16p11.2 DEL mice	Kumar et al., 2018 (PMID: 29844452)
TBX6	T-box 6	602427	TBX6-dependent regulation of SOX2 is involved in the specification of paraxial mesoderm	16p11.2 DUP patients and mice have increased risk of congenital vertebral malformations	Ren et al., 2020 (PMID: 31888956)
				Heterozygous loss of function of TBX6 in humans and mice is associated with congenital anomalies of the kidney and urinary tract (CAKUT)	Yang et al., 2020 (PMID: 32450157)
YPEL3	Yippee-like 3 (*Drosophila*)	609724	Member of putative Zinc-finger motif containing proteins, p53-regulated tumour suppressor	YPEL3 loss of function is linked with abnormal brain morphology in Zebrafish	Blaker-Lee et al., 2012 (PMID: 22566537)
GDPD3	Glycerophosphodiester phosphodiesterase domain- containing protein 3	616318	Lysophospholipase activity	GDPD3 loss of function produces abnormal brain morphology in Zebrafish	Blaker-Lee et al., 2012 (PMID: 22566537)
MAPK3	Mitogen-activated protein kinase 3	601795	Protein kinase implicated in cell proliferation, synaptic and behavioural plasticity	Loss of function of MAPK3 produces abnormal body length, deficient axon tracts and defective neural tubes in Zebrafish	Blaker-Lee et al., 2012 (PMID: 22566537)
				MAPK3 knock-down causes reductions in climbing ability in *Drosophila* and seizure phenotype; the knock-out is associated with aberrant axonal-synaptic targeting	Iyer et al., 2018 (PMID: 29959322)
				ERK1 is hyperphosphorylated in male 16p11.2 DEL mice during reward learning	Grissom et al., 2018 (PMID: 29038598)
				ERK1/2 activity is elevated in 16p11.2 DEL mice. Treatment with Ras-ERK inhibitors rescues cortical cytoarchitectural and behavioural deficits	Pucilowska et al., 2018 (PMID: 29934348)
CORO1a	Coronin, actin-binding protein 1A	605000	T lymphocyte trafficking and survival	Correlates with reduced lymphocytes count in 16p11.2 DEL patients presenting a concomitant low dosage of BOLA2 duplicone	Giannuzzi et al., 2022 (PMID: 35715439)
				CORO1a loss of function causes abnormal body length, defective neural tube, deficient axon tracts and movement defects in Zebrafish	Blaker-Lee et al., 2012 (PMID: 22566537)
				Enriched in Drd2+ MSNs in 16p11.2 DEL mice	Portmann et al., 2014 (PMID: 24794428)

So far, 3 mouse models for 16p11.2 deletion (DEL) have been generated, carrying the heterozygous deletion in the Slx1b-Sept1 region (Mills model) (Horev et al., 2011), in the Coro1a-Spn region (Dolmetsch model) (Portmann et al., 2014) or in the Sult1a1-Spn region (Herault model) (Arbogast et al., 2016). Two mouse models for 16p11.2 duplication (DUP) are currently available, carrying the heterozygous duplication in the Slx1b-Sept1 region (Mills model) (Horev et al., 2011) or in the Sult1a1-Spn region (Herault model) (Arbogast et al., 2016). Despite the presence of similar phenotypes, these mouse models also show some differences which might be partly explained by the different targeting regions on chromosome 7, as highlighted in (Arbogast et al., 2016). Here and in Table 2, we summarize the main phenotypes observed in DEL and DUP animal models.

**TABLE 2:** Main phenotypes of 16p11.2 CNVs mouse models.

Model	Engineered region	Phenotypes	References
Mills deletion	Slx1b-Sept1	**Neuroanatomical and metabolic phenotypes**	Horev et al., 2011 (PMID 21969575); Pucilowska et al., 2015 (PMID 25698753)
		Decreased brain size and cortical thickness	
		Reduction of upper cortical layers neurons	Pucilowska et al., 2015 (PMID 25698753); Pucilowska et al., 2018 (PMID 29934348)
		Increased volumes of midbrain, hypothalamus, striatum, nucleus accumbens, globus pallidus and cerebellar cortex	Horev et al., 2011 (PMID 21969575); Pucilowska et al., 2018 (PMID 29934348)
		Decreased volumes of ventral hippocampus, lateral septum, amygdala and enthorinal cortex	Pucilowska et al., 2018 (PMID 29934348)
		Early post-natal mortality; pups show lower body weight	Horev et al., 2011 (PMID 21969575)
		**Signalling alterations**	Grissom et al., 2018 (PMID 29038598)
		Males overexpress mRNA for D2 receptors and adenosine 2a receptor in the striatum	
		**Neurophysiological phenotypes**	Wang et al., 2018 (PMID 29853627)
		Deficient NMDAR-mediated glutamatergic transmission in the mPFC	
		Compromised connectivity on the orbitofrontal, insular and auditory axis, and between the septum and hippocampus	Openshaw et al., 2023 (PMID 37225770)
		**Behavioural phenotypes**	Horev et al., 2011 (PMID 21969575); Angelakos et al., 2017 (PMID 27739237)
		Higher locomotor activity in familiar environments and stereotyped behaviours	
		Initial hypoactivity in novel environments	Pucilowska et al., 2015 (PMID 25698753)
		Deficits in righting from upside-down position	Brunner et al., 2015 (PMID 26273832)
		Impairments in spatial memory	Wang et al., 2018 (PMID 29853627)
		Deficits in novel object recognition	Pucilowska et al., 2015 (PMID 25698753); Pucilowska et al., 2018 (PMID 29934348)
		Deficits in passive avoidance	Tian et al., 2015 (PMID 25581360)
		Deficits in contextual fear conditioning	Tian et al., 2015 (PMID 25581360); Pucilowska et al., 2018 (PMID 29934348)
		Deficits in pre-pulse inhibition, but enhanced performance in attentional tasks	Openshaw et al., 2023 (PMID 37225770)
		Reduced ultrasonic vocalization during male-female interaction	Stoppel et al., 2017 (PMID 28984295)
		In C57/Bl6 pure background, normal social behaviour in the three-chamber test	Brunner et al., 2015 (PMID 26273832)
		Male-specific deficits in perinatal communication	Agarwalla et al., 2020 (PMID 32558237)
		Male-specific sleep deficits	Angelakos et al., 2017 (PMID 27739237)
		Male-specific deficits in reward learning	Grissom et al., 2018 (PMID 29038598)
Dolmetsch deletion	Coro1a-Spn	**Neuroanatomical and metabolic phenotypes**	Portmann et al., 2014 (PMID 24794428)
		Increased volumes of midbrain, hypothalamus, striatum, nucleus accumbens, globus pallidus and cerebellar cortex	
		Early post-natal mortality; pups show lower body weight	Portmann et al., 2014 (PMID 24794428)
		**Signalling alterations**	Portmann et al., 2014 (PMID 24794428)
		Increased number of striatopallidal MSNs in the striatum; decreased number of striatonigral MSNs in the cortex and DARPP-32 expressing neurons; increased number of MSNs co-expressing D1 and D2 receptors	
		**Neurophysiological phenotypes**	Portmann et al., 2014 (PMID 24794428)
		Increased ratio of AMPA to NMDA receptor-mediated EPSC and increased miniature EPSC frequency	
		**Behavioural phenotypes**	Portmann et al., 2014 (PMID 24794428)
		Lack of gait fluidity and tremor	
		Higher locomotor activity in familiar environments and stereotyped behaviours	Portmann et al., 2014 (PMID 24794428); Yang et al., 2015 (PMID 25663600)
		Initial hypoactivity in novel environments	Portmann et al., 2014 (PMID 24794428)
		Deficits in novel object recognition	Portmann et al., 2014 (PMID 24794428); Yang et al., 2015 (PMID 26572653)
		Deficits in novel object location and touchscreen pairwise visual discrimination acquisition and reversal. Unaffected contextual fear conditioning	Yang et al., 2015 (PMID 26572653)
		Reduced ultrasonic vocalization during male-female interaction	Yang et al., 2015 (PMID 25663600)
		In C57/Bl6 pure background, normal social behaviour in the three-chamber test	Yang et al., 2015 (PMID 26066718)
		Impairments in recognition and ultrasonic vocalization are displayed only in standard mixed-genotype housing conditions	Yang et al., 2015 (PMID 26066718)
Herault deletion	Sult1a-Spn	**Neuroanatomical and metabolic phenotypes**	
		Decreased skull size and altered skull shape in females	Arbogast et al., 2016 (PMID 26872257)
		Early post-natal mortality; pups and and adults show lower body weight. Higher energy expenditure during dark phase, faster glucose clearance and lower levels of leptin and adiponectin	Arbogast et al., 2016 (PMID 26872257)
		**Gene expression alterations**	Arbogast et al., 2016 (PMID 26872257)
		Gene expression dysregulation, especially in the striatum	
		**Neurophysiological phenotypes**	Arbogast et al., 2016 (PMID 26872257)
		No alterations in hippocampal excitability	
		**Behavioural phenotypes**	Arbogast et al., 2016 (PMID 26872257)
		Higher locomotor activity in familiar environments and stereotyped behaviours; normal motor coordination	
		Deficits in novel object recognition	Arbogast et al., 2016 (PMID 26872257)
		Social deficits in the three chamber test only in a mixed C57/Bl6N X C3B background	Arbogast et al., 2016 (PMID 26872257)
Mills duplication	Slx1b-Sept1	**Neuroanatomical phenotypes**	Horev et al., 2011 (PMID 21969575)
		Trend toward reduced brain volumes in several brain regions	
		**Neurophysiological phenotypes**	Rein et al., 2020 (PMID 32099100)
		Hypexcitability of mPFC neurons due to impaired GABAergic transmission. Unchanged glutamatergic transmission	
		**Gene expression alterations**	Rein et al., 2020 (PMID 32099100)
		Gene expression dysregulation in mPFC neurons, with downregulation of Npas4	
		**Behavioural phenotypes**	Horev et al., 2011 (PMID 21969575); Rein et al., 2020 (PMID 32099100)
		Hypolocomotion in novel environments	
		In females, hypolocomotion is displayed during home-cage monitoring, while in males it is displayed in novel environments	Bristow et al., 2020 (PMID 32320645)
		Anxiety behaviour in males	Bristow et al., 2020 (PMID 32320645)
		Female-specific reduction of pre-pulse inhibition	Bristow et al., 2020 (PMID 32320645)
		No deficits in pre-pulse inhibition	Rein et al., 2020 (PMID 32099100)
		Deficits in social approach and in three-chamber test	Rein et al., 2020 (PMID 32099100)
		Reduced time spent in proximity of cage-mates	Bristow et al., 2020 (PMID 32320645)
		Impairments in spatial working memory, slower learning and more impulsive responding in the continuous performance task	Bristow et al., 2020 (PMID 32320645)
		Deficits in temporal order recognition memory	Rein et al., 2020 (PMID 32099100)
		Unaffected novel object recognition	Rein et al., 2020 (PMID 32099100)
Herault duplication	Sult1a-Spn	**Neuroanatomical and metabolic phenotypes**	Arbogast et al., 2016 (PMID 26872257)
		Altered skull shape, but no differences in skull size	
		Increased body weight, with lower energy expenditure during light and dark phase, lower glucose clearance and higher levels of leptin	Arbogast et al., 2016 (PMID 26872257)
		**Behavioural phenotypes**	Arbogast et al., 2016 (PMID 26872257)
		Hypolocomotion	
		Social deficits in the three chamber test only in a mixed C57/Bl6N X C3B background	Arbogast et al., 2016 (PMID 26872257)
		Enhanced novel object recognition	Arbogast et al., 2016 (PMID 26872257)

### 16p11.2 DEL mouse models

#### 16p11.2 DEL mice display metabolic and neuroanatomical alterations

All DEL mouse models are affected by early post-natal mortality and their body weight is significantly lower than wild-type mice (Horev et al., 2011; Portmann et al., 2014; Arbogast et al., 2016). In Dolmetsch model, this effect could be corrected by improved nutrition and separation from wild-type littermates (Portmann et al., 2014). As adults, the body weight of DEL mice is similar to wild-type littermates (Horev et al., 2011) or reduced (Portmann et al., 2014; Pucilowska et al., 2015; Arbogast et al., 2016), showing decreased adiposity (Arbogast et al., 2016). This is in contrast with human findings where the 16p11.2 deletion has been associated with a highly penetrant type of obesity (Jacquemont et al., 2011). Herault model also displays a higher energy expenditure during the dark phase, a faster glucose clearance and lower blood levels of leptin and adiponectin (Arbogast et al., 2016).

Craniofacial dysmorphisms have been reported by (Arbogast et al., 2016), with decreased skull size and altered skull shape in the Herault DEL females. In contrast with humans (Qureshi et al., 2014), the brain size is modestly reduced in Mills DEL mice during early post-natal development (Pucilowska et al., 2015). This phenotype was observed also in Mills DEL adults (Horev et al., 2011; Pucilowska et al., 2015), with no significant changes in the grey matter (Kumar et al., 2018). In addition, cortical thickness is decreased in Mills DEL mice (Pucilowska et al., 2015), similarly to human carriers (Maillard et al., 2015). In particular, this model shows an aberrant cortical cytoarchitecture, with a reduction of upper cortical layer neurons at embryonic day 14.5, probably due to aberrant progenitor proliferation and premature cell cycle exit, leading to depletion of progenitor pools (Pucilowska et al., 2015; Pucilowska et al., 2018). Despite the general reduction in brain size, Mills and Dolmetsch DEL mice show increases in the relative volumes of several brain areas, including the midbrain, hypothalamus, striatum, nucleus accumbens, globus pallidus and cerebellar cortex (Horev et al., 2011; Portmann et al., 2014; Pucilowska et al., 2018). Mills DEL mice also exhibit decreased volumes in the ventral hippocampus, lateral septum, amygdala and entorhinal cortex (Pucilowska et al., 2018).

#### 16p11.2 DEL mice show dopaminergic signalling dysregulation

Interestingly, data obtained in both Dolmetsch and Mills models suggest major alterations in dopaminergic signalling. For instance, Portmann et al. observed a significant increase in medium spiny neurons expressing dopamine receptor 2 (Drd2+ MSNs) in the striatum of 16p11.2 DEL neonates, with no changes in medium spiny neurons expressing dopamine receptor 1 (Drd1+ MSNs) (Portmann et al., 2014). The observed increase in striatopallidal MSNs in the striatum resulted in a reduced sensitivity to sedation induced by risperidone, a D2 receptor antagonist (Portmann et al., 2014). Interestingly, an increased number of cells co-expressing Drd1 and Drd2 was observed, suggesting that the 16p11.2 deletion may also affect the process of MSNs specification. In the deep layers of the cortex, Drd1+ MSNs were significantly decreased as well as DARPP-32 expressing neurons. Moreover, tyrosine-hydroxylase (TH), a rate limiting enzyme in the dopamine (DA) synthesis pathway, was decreased in mesodiencephalic DA cells (Portmann et al., 2014). Imbalances in the ratio of D1 and D2-expressing MSNs were also observed in adult brains by Grissom et al. In particular, Mills DEL males, but not females, overexpressed the mRNA for D2 receptor and adenosine 2a receptor in the striatum (Grissom et al., 2018). Interestingly, transcriptomic analysis in Herault DEL model revealed dysregulations in gene expression in DEL mice in different brain regions, with the striatum being more severely impacted (Arbogast et al., 2016). This finding may explain some of the motor and cognitive deficits displayed by these animals, dependent on basal ganglia circuitry.

#### 16p11.2 DEL mice display significant changes in the excitability profile of MSNs and cortical pyramidal neurons, as well as compromised connectivity between different brain regions

The presence of major alterations in dopamine-mediated circuits is further supported by electrophysiological recordings on striatal MSNs from Dolmetsch DEL mice. These studies revealed an increased ratio of AMPA to NMDA receptor-mediated excitatory post synaptic currents (EPSC) and an increased miniature EPSC (mEPSC) frequency. Conversely, the paired-pulse ratios (PPRs) across multiple interstimulus intervals (ISIs) were significantly decreased (Portmann et al., 2014). These results suggest that the release probability of excitatory synapses on MSNs may be augmented.

In the hippocampus, no alterations were found in the excitability profile in Herault DEL model (Arbogast et al., 2016). However, in the medial prefrontal cortex (mPFC) of Mills DEL mice, pyramidal neurons displayed deficient NMDA-receptor-mediated glutamatergic transmission and reduced frequency of action potential (AP) firing (Wang et al., 2018). Compromised functional connectivity on the orbitofrontal, insular and auditory axis, and between the septum and the hippocampal regions, has been also reported in Mills DEL mice (Openshaw et al., 2023).

#### 16p11.2 DEL mice recapitulates some of the behavioural deficits affecting human carriers

At the behavioural level, mild motor impairments have been observed in 16p11.2 DEL mice, such as deficits in righting from upside-down position (Mills DEL model) (Brunner et al., 2015) and lack of gait fluidity and tremor (Dolmetsch DEL model) (Portmann et al., 2014). However, normal motor coordination in the rotarod test was reported in Herault DEL model (Arbogast et al., 2016). Higher locomotor activity in familiar environments and stereotyped behaviours have also been broadly reported in all models (Horev et al., 2011; Portmann et al., 2014; Yang et al., 2015c; Arbogast et al., 2016; Angelakos et al., 2017). However, when tested in novel environments, such as in the open field test, both Dolmetsch and Mills DEL mice showed initial hypoactivity that gradually disappeared over the course of the first 10 min (Portmann et al., 2014; Pucilowska et al., 2015) which might reflect deficits in motor initiation (Portmann et al., 2014) or increased anxiety, as shown by Pucilowska et al., in the elevated plus maze test (Pucilowska et al., 2015).

DEL mice also display a wide range of cognitive deficits, including impairments in spatial memory (Mills DEL model) (Wang et al., 2018), novel object recognition (Mills, Dolmetsch and Herault DEL models) (Portmann et al., 2014; Yang et al., 2015b; Pucilowska et al., 2015; Arbogast et al., 2016; Pucilowska et al., 2018), novel object location (Dolmetsch DEL model) (Yang et al., 2015b) and passive avoidance (Mills DEL model) (Tian et al., 2015). Dolmetsch DEL model also exhibited prominent cognitive impairments in the touchscreen pairwise visual discrimination acquisition and reversal (Yang et al., 2015b), while deficits in contextual fear conditioning have been observed in Mills DEL model (Tian et al., 2015; Pucilowska et al., 2018), but not in Dolmetsch DEL model (Yang et al., 2015b). Consistently with the impaired fronto-temporal connectivity and GABAergic dysfunction observed by Openshaw et al., Mills DEL mice showed deficits in pre-pulse inhibition (PPI), a measure of sensorimotor gating, but enhanced performance in attentional tasks (Openshaw et al., 2023).

In terms of social behaviour, ultrasonic vocalizations during male-female interactions are significantly reduced in DEL mice (Mills and Dolmetsch DEL models) (Yang et al., 2015c; Stoppel et al., 2018), that could be rescued upon chronic activation of GABA_B_ receptors (Stoppel et al., 2018). Unexpectedly, all DEL models display normal behaviour in the three-chamber social preference test, in contrast with the social deficits affecting human carriers (Yang et al., 2015a; Brunner et al., 2015; Arbogast et al., 2016). This could be partially due to the aberrant increase of oxytocin levels exhibited by the Mills DEL mice (Pucilowska et al., 2018) that may mask potential social deficits. In addition, the genetic background can profoundly influence the manifestation of social impairments. As demonstrated by Arbogast et al., a significant decrease in social preference for the second stranger in the three-chamber test can be observed in Herault DEL model with a hybrid C57/Bl6N X C3B background, but not in mice with a pure C57/Bl6N background (Arbogast et al., 2016). Housing conditions also seem to affect the emergence of social and cognitive deficits. For instance, impairments in recognition memory and ultrasonic vocalisation are displayed by Dolmetsch DEL mice only in standard mixed-genotypes housing conditions, but not by animals housed with individuals of the same genotype (Yang et al., 2015a).

Despite the higher prevalence of autism spectrum disorder in males, sex-specific phenotypes have not been systematically investigated in DEL mice. Kumar et al. found prominent male-specific structural changes in medial fibre tracts proximate to the striatum, overlapping with specific gene expression patterns associated with neurite outgrowth and MAPK pathway (Mills DEL model) (Kumar et al., 2018). At the behavioural level, male-specific deficits in perinatal communication have been observed (Mills DEL model) (Agarwalla et al., 2020). Consistently with ASD and ADHD patients’ phenotypes, male-specific sleep/wake decrements in total sleep time and longer bouts of continuous wakefulness have been reported (Mills DEL model) (Angelakos et al., 2017). Moreover, Mills DEL males display reduced motivation and impaired reward learning, which is consistent with the observed increase in the mRNA coding for dopamine D2 receptors associated with behavioural inhibition (Grissom et al., 2018).

### 16p11.2 DUP mouse models

#### 16p11.2 DUP mice show opposite metabolic and neuroanatomical phenotypes in comparison with the 16p11.2 DEL mice

In comparison with DEL mice, DUP mice show opposite phenotypes in terms of body weight and metabolism, with increased body size, lower energy expenditure during the light and dark phase, a lower glucose clearance and higher blood levels of leptin (Herault model) (Arbogast et al., 2016). Craniofacial dysmorphisms have also been observed in DUP mice, showing altered skull shape but no changes in the skull size (Herault model) (Arbogast et al., 2016). At the neurostructural level, DUP mice are not significantly different from wild types, although a trend toward reduced volumes in several brain regions can be observed (Mills model) (Horev et al., 2011).

#### 16p11.2 DUP mice display some behavioural deficits reminiscent of those observed in human carriers

In contrast with DEL mice, both Herault and Mills DUP mice display hypolocomotion (Horev et al., 2011; Arbogast et al., 2016; Bristow et al., 2020; Rein et al., 2020) with potential sex-differences. Hypo locomotion was observed in Mills DUP females only during home cage monitoring whereas males exhibited this behaviour in the novel environment of the open field arena. This observation may indicate that additional factors, such as the levels of stress, may have an impact on sex-specific phenotypes. These results are also consistent with the increased anxiety behaviour observed only in males, possibly linked to the reduced hippocampal-orbitofrontal-amygdala connectivity (Bristow et al., 2020). Importantly, this circuitry has been implicated in thought disorder, a hallmark of schizophrenia (Sumner et al., 2018). In Mills DUP mice, typical phenotypes linked to schizophrenia, such as MK-801-induced hyperlocomotion and deficits in pre-pulse inhibition, were not observed by Rein et al. (Rein et al., 2020), although there is a report of female-specific reduction of pre-pulse inhibition (Bristow et al., 2020). Mills DUP model also displays social impairments reminiscent of ASD, including deficits in social approach and in the three-chamber test (Rein et al., 2020) as well as reduced time spent in proximity with cage mates (Bristow et al., 2020). In contrast, in Herault DUP model, social deficits in the three-chamber test could be observed only in the hybrid C57/Bl6N X C3B background, similarly to the DEL mice (Arbogast et al., 2016).

Consistently with the observed deficits in hippocampal-orbitofrontal-amygdala connectivity, Mills DUP mice display impairments in spatial working memory (Bristow et al., 2020), that have been also reported in DUP human carriers (Schobel et al., 2009). Similarly to the deficits observed in patients in the continuous performance task (Fleck et al., 2001), Mills DUP mice show slower learning and more impulsive responding (Bristow et al., 2020), as well as prefrontal cortex-dependent cognitive impairments in the temporal order recognition memory (Rein et al., 2020). Novel object recognition memory was either found unaffected by the CNV (Rein et al., 2020) or significantly enhanced (Arbogast et al., 2016), possibly due to the different models and experimental protocols used in these studies.

#### 16p11.2 DUP mice display major GABAergic dysfunctions, mediated by the transcription factor Npas4

In contrast with DEL mice showing hypoactivity in the mPFC neurons, Mills DUP mice display hyperexcitability due to a significant impairment in GABAergic synaptic transmission, while glutamatergic transmission was found unchanged (Rein et al., 2020). This is consistent to the excitatory/inhibitory imbalance observed in ASD patients (Nelson and Valakh, 2015).

In order to determine the effect of the 16p11.2 duplication on gene expression, Rein et al. performed RNA-sequencing on mPFC and identified 388 differentially expressed genes, most of which were downregulated, including epigenetic markers, ASD/ID risk genes and the sodium ion channel SCN9a. A significant downregulation was detected for Npas4, a transcription factor promoting the formation of GABAergic synapses, that was found reduced also in post-mortem PFCs from idiopathic ASD patients (Rein et al., 2020). Restoration of Npas4 levels in Mills 16p11.2 DUP mice was sufficient to rescue the synaptic and behavioural deficits, thus suggesting the pathogenic role of Npas4 in the GABAergic dysfunction underlying the 16p11.2 DUP phenotype (Rein et al., 2020).

### Other animal models

#### 16p11.2 DEL and DUP rat models

Rat models for 16p11.2 CNVs have also been generated recapitulating craniofacial phenotypes, with mirror effects between the DEL and the DUP conditions (Qiu et al., 2019). Converging and male-specific deficits in social behaviour and novel object recognition have been also observed in 16p11.2 DEL and DUP rats in two different genetic backgrounds (Martin Lorenzo et al., 2023). RNA sequencing analysis in the hippocampus found 267 differentially expressed genes dysregulated in DEL and DUP rat models. Among these genes, 100 were downregulated and 120 upregulated in both models, which could explain some overlapping phenotypes, independent from gene dosage. Differential functional analysis revealed 23 upregulated pathways in both DEL and DUP rats, associated with morphogenesis of the primary cilium. However, pathways related to synaptic function and metabolism were mostly deregulated in DEL rats, while pathways associated with transcription, epigenomic regulation and hormone regulation were mostly affected in DUP animals (Martin Lorenzo et al., 2023).

Recently, Yang et al. carried out anatomical and electrophysiological analysis of developing interneurons in 16p11.2 DEL rats, after the identification of a subset of interneurons in human foetal cerebral cortex potentially vulnerable to genetic autism risk factors. In 16p11.2 DEL rats at P21, the number or position of INs was unchanged in either CA1 or somatosensory cortex. However, somatostatin-expressing INs in CA1 display hyperexcitability, with an enlarged axon initial segment. This finding, although limited to a single developmental stage and one type of INs, supports the idea that the 16p11.2 deletion may perturb the electrophysiological properties of developing INs, thereby affecting the excitation/inhibition (E/I) balance (Yang et al., 2023).

### Zebrafish and *Drosophila melanogaster* models

A deeper characterization of the effects of 16p11.2 CNVs at the cellular level has been achieved using more simplified animal models, allowing a high-throughput analysis of the interaction between genetic and phenotypic effects. For instance, the first 5 days of development in Zebrafish recapitulate the first weeks of development in mice and the first couple of years in humans, thus allowing to detect abnormalities in brain structures or functions that become obvious only after birth (Blaker-Lee et al., 2012). Among the 27 protein-coding genes in the 16p11.2 region, 21 are also present in Zebrafish. This model has been used to discover dosage-sensitive genes within the 16p11.2 locus by selective induced loss of function (LOF) or overexpression experiments. By performing LOF studies from 24 h post-fertilization to post-natal day 5, covering a period from 5-week gestation to toddlerhood in humans, Blaker-Lee et al. revealed that most of the 16p11.2 genes are highly active during early development and are involved in brain and body development. Selective LOF of 16p11.2 homologs was associated with spontaneous movement defects and reduced or no response to touch, possibly linked to the observed abnormalities in axonal development (Blaker-Lee et al., 2012). Using similar approaches, KCTD13 (see Table 1) was identified as a major driver of head size phenotypes, which were consistent with those observed in DEL and DUP human carriers. In particular, KCTD13 suppression induced macrocephaly in Zebrafish embryos resembling the 16p11.2 DEL condition, whereas its overexpression caused microcephaly with concomitant defects in neurogenesis, in epistasis with two other genes in the locus, MAPK3 and MVP (Golzio et al., 2012). In addition, a genetic interaction was demonstrated between KCTD13 and ciliopathy-associated genes (Migliavacca et al., 2015).

*Drosophila melanogaster*, which has at least 14 homologs of human 16p11.2 genes, has also been employed to test the role of these individual genes and their combinatorial effects in determining the variegated phenotypes observed in DEL and DUP human carriers (Park et al., 2016; Iyer et al., 2018). By genetic screening and RNA interference approaches, KIF22, a member of kinesin family (see Table 1), was identified as a key factor required for the establishment of synaptic connectivity in *Drosophila* neuromuscular junction (Park et al., 2016). By performing knock-down of single homologs and 564 pairwise knockdowns, Iyer et al. identified 24 interactions between 16p11.2 homologs and 46 interactions between 16p11.2 homologs and neurodevelopmental genes. In particular, they observed impaired motor functions and spontaneous seizures, as well as alterations in the architecture of *Drosophila* neuromuscular junction and dendritic arborization, consistently with the phenotypes observed in human carriers. Moreover, several homologs contributed in different proportion to the cellular composition of the fly eye, probably intervening at different timepoints during cellular proliferation and differentiation. In general, the data from Iyer et al. suggest that several genes within the 16p11.2 region are involved in neurodevelopment and their reciprocal interaction is responsible for the heterogeneous phenotypes observed in patients (Iyer et al., 2018).

## Cellular models of 16p11.2 deletion and duplication

Recent advances in stem cell technologies opened the possibility to investigate neurodevelopmental disorders using patient-derived induced pluripotent stem cells (iPSCs). For instance, Deshpande et al. used iPSCs-derived forebrain cortical neurons to investigate the cellular mechanisms underlying differences in brain size associated with 16p11.2 CNVs. DEL neural progenitors display increased soma size and dendritic length as well as a more extensive arborization. In contrast, DUP neurons show opposing phenotypes. The larger neuronal size in the DEL condition was associated to altered functional properties, such as reduced excitability and membrane resistance, while the DUP neurons did not significantly deviate from controls. Interestingly, DUP neurons displayed increased outward potassium current, to stabilize their intrinsic excitability. Both DEL and DUP neurons showed less excitatory synapses with increased synaptic strength, that may underlie the altered network activity and behavioural deficits in human carriers (Deshpande et al., 2017).

Neuronal firing and synchrony have been found reduced in iPSCs-derived excitatory neurons harbouring the 16p11.2 duplication, in later stages of development, along with reduced dendrite length and impaired calcium homeostasis (Parnell et al., 2023). These findings were recapitulated in excitatory neurons derived from DUP patients with schizophrenia, thus linking excitatory neurons dysfunctions with schizophrenia pathogenesis. Transcriptomic analysis carried out on excitatory neurons after 7 weeks maturation identified 62 upregulated and 133 downregulated genes, associated with calcium ion binding and neuron projections development (Parnell et al., 2023).

Similarly to cortical neurons, iPSCs-derived dopaminergic neurons from DEL patients also displayed increased soma size. However, in contrast with the cortical neurons’ phenotype, these morphological changes correlated with increased hyperexcitability of DEL dopaminergic neurons. Interestingly, DEL dopaminergic neurons show reduced levels of KCDT13 and overexpression of RHOA, a molecular pathway also upregulated in KCDT13 heterozygous and in 16p11.2 DEL mice (Escamilla et al., 2017; Martin Lorenzo et al., 2021). Treatment with RHOA inhibitor could rescue the cell size and hyperexcitability of DEL dopaminergic neurons, thus implicating RHOA pathway in dopaminergic network excitability (Sundberg et al., 2021).

Macrocephaly in DEL carriers was recently found associated with hyperproliferation of iPSCs-derived neural progenitors, that was inversely correlated with ERK1/2 phosphorylation and response to basic fibroblast growth factor (bFGF), a mitogen that activates ERK pathway (Connacher et al., 2022). In contrast, two previous studies did not detect any differences in cell proliferation at the early stage of cortical progenitors (Deshpande et al., 2017; Sundberg et al., 2021). Brain overgrowth in the 16p11.2 deletion syndrome has been potentially linked to overexpression of CD47 in both neural and oligodendrocyte progenitor cells. CD47 is a “do not eat me” signal protein, thus preventing cells from getting engulfed or phagocytosed by macrophages and microglia (Li et al., 2021).

To date, two studies employed cerebral organoids to investigate the effects of the 16p11.2 deletion on brain development (Urresti et al., 2021; Fetit et al., 2023). Importantly, DEL and DUP cortical organoids could recapitulate the brain size phenotypes. In addition, DEL cortical organoids exhibited increased neuronal maturation, soma size and neurite length as well as depletion of neural progenitors, in comparison with control and DUP organoids. However, neuronal migration was significantly impaired in both DEL and DUP organoids. When looking at KCTD3 and total RHOA levels, the authors found decreased KCTD3 and increased RHOA levels in DEL organoids, while the DUP organoids showed opposite trends. However, the active GTP-bound form of RHOA was consistently upregulated in both CNVs, that was previously linked with impaired neuronal migration (Cappello et al., 2012). Consistently, the observed defects in neuronal migration in both DEL and DUP organoids could be rescued by RHOA inhibition (Urresti et al., 2021).

The effects of 16p11.2 CNVs on interneurons development was recently investigated by Fetit et al. Ventral organoids harbouring the 16p11.2 deletion were more variable in size compared with the isogenic controls (Fetit et al., 2023). This variability could be relevant when considering the clinical heterogenicity of DEL human carriers (Fetit et al., 2020). In addition, the authors found a substantial acceleration of subpallial development in DEL organoids, potentially leading to premature differentiation (Fetit et al., 2023).

## Role of the 16p11.2 genes

The purpose of this section is to review the specific functions of the genes within the 16p11.2 region and how they might interact in specific molecular pathways, thus determining the phenotypic effects observed in patients, animal, and cellular models. As listed in Table 1, 27 protein-coding genes are involved in 16p11.2 CNVs. Some of them have been well characterised in animal and cellular models created to recapitulate the phenotypes observed in DEL and DUP patients. Other 16p11.2 genes still have unknown function, and their role in the pathogenesis of 16p11.2-associated diseases is obscure. We will describe a small subset of genes clearly involved in the 16p11.2 associated phenotypes (MAPK3, KDCT13, MVP, TAOK2 and SEZ6l2) and thus potential therapeutic targets. Subsequently, we will describe the 16p11.2 genes with known cellular functions, but not yet linked to the 16p11.2 DEL and DUP pathologies. Then, we will briefly list the limited information available for the least characterised genes. Finally, we will consider non-coding RNAs that are located within the 16p11.2 region but have not yet been linked to specific cellular functions (see Table 3; Figure 1).

**TABLE 3:** List of non-coding RNAs mapping on the 16p11.2 region.

*Non-coding RNA*	*Length*	*Start-end locations on chromosome 16 (basepairs)*	*Proximity to genes in the locus*
microRNA hsa-mir-3680 precursor (hsa-mir-3680–1, hsa-mir-3680–2)	*87 nucleotides*	*21,506,049–21,506,135* *29,599,179–29,599,265*	*Next to SPN*
**hsa-miR-3680-3p**	*22 nucleotides*		*Next to SPN*
**hsa-miR3680-5p**	*21 nucleotides*		*Next to SPN*
HSALNT0385266Possible ORF	*1921 nucleotides*	*29,862,659–29,868,081*	*Between CDIPT and SEZ6L2*
HSALNT0385275	*746 nucleotides*	*29,862,659–29,868,120*	*Between CDIPT and SEZ6L2*
HSALNT0385268Possible ORF	*1649 nucleotides*	*29,862,849–29,868,081*	*Between CDIPT and SEZ6L2*
HSALNT0385269Possible ORF	*1078 nucleotides*	*29,862,849–29,868,048*	*Between CDIPT and SEZ6L2*
HSALNT0385276	*893 nucleotides*	*29,863,289–29,868,050*	*Between CDIPT and SEZ6L2*
HSALNT0385277	*855 nucleotides*	*29,863,289–29,868,050*	*Between CDIPT and SEZ6L2*
HSALNT0385278	*938 nucleotides*	*29,863,292–29,868,051*	*Between CDIPT and SEZ6L2*
HSALNT0229585	*629 nucleotides*	*29,863,336–29,868,048*	*Between CDIPT and SEZ6L2*
non-protein coding lnc-MVP-3:1Possible ORF	*491 nucleotides*	*29,863,551–29,865,434*	*Between CDIPT and SEZ6L2*
non-protein coding lnc-MVP-3:2	*620 nucleotides*	*29,863,551–29,868,050*	*Between CDIPT and SEZ6L2*
non-protein coding lnc-MVP-3:3Possible ORF	*652 nucleotides*	*29,863,578–29,868,048*	*Between CDIPT and SEZ6L2*
non-protein coding lnc-MVP-3:4Possible ORF	*1025 nucleotides*	*29,863,578–29,868 050*	*Between CDIPT and SEZ6L2*
non-protein coding lnc-MVP-3:5Possible ORF	*741 nucleotides*	*29,863,578–29,868,050*	*Between CDIPT and SEZ6L2*
non-protein coding lnc-MVP-3:7Possible ORF	*726 nucleotides*	*29,863,593–29,868,050*	*Between CDIPT and SEZ6L2 Between CDIPT and SEZ6L2*
non-protein coding lnc-MVP-3:8Possible ORF	*729 nucleotides*	*29,863,593–29,868,053*	*Between CDIPT and SEZ6L2*
**HSALNT0229588**	*845 nucleotides*	*29,863,614–29,868,048*	*Between CDIPT and SEZ6L2*
**HSALNT0229589**	*949 nucleotides*	*29,863,614–29,868,048*	*Between CDIPT and SEZ6L2*
non-protein coding lnc-MVP-3:9	*589 nucleotides*	*29,863,674–29,867,994*	*Between CDIPT and SEZ6L2*
non-protein coding lnc-MVP-3:10	*785 nucleotides*	*29,863,674–29,868,048*	*Between CDIPT and SEZ6L2*
non-protein coding lnc-MVP-3:11	*495 nucleotides*	*29,863,674–29,868,048*	*Between CDIPT and SEZ6L2*
non-protein coding lnc-MVP-3:12	*891 nucleotides*	*29,863,674–29,868,050*	*Between CDIPT and SEZ6L2*
non-protein coding lnc-MVP-3:13	*639 nucleotides*	*29,863,683–29,868,053*	*Between CDIPT and SEZ6L2*
CDIP transferase opposite strand, pseudogene, transcript variant 1 (CDIPTOSP)	*782 nucleotides*	*29,863,683–29,868,053*	*Between CDIPT and SEZ6L2*
HSALNT0385279	*582 nucleotides*	*29,863,684–29,868,035*	*Between CDIPT and SEZ6L2*
HSALNT0385280	*639 nucleotides*	*29,863,789–29,868,120*	*Between CDIPT and SEZ6L2*
CDIP transferase opposite strand, pseudogene, transcript variant 2 (CDIPTOSP)	*659 nucleotides*	*29,863,834–29,868,053*	*Between CDIPT and SEZ6L2*
**N** on-protein coding lnc-MVP-3:14	*756 nucleotides*	*29,863,834–29,868,047*	*Between CDIPT and SEZ6L2*
non-protein coding lnc-MVP-3:15	*762 nucleotides*	*29,863,834–29,868,053*	*Between CDIPT and SEZ6L2*
non-protein coding lnc-MVP-3:16	*705 nucleotides*	*29,863,847–29,868,047*	*Between CDIPT and SEZ6L2*
HSALNT0385274	*529 nucleotides*	*29,863,848–29,868,020*	*Between CDIPT and SEZ6L2*
non-protein coding lnc-MVP-3:18	*738 nucleotides*	*29,863,852–29,868,047*	*Between CDIPT and SEZ6L2*
non-protein coding lnc-MVP-3:19	*852 nucleotides*	*29,863,852–29,868,048*	*Between CDIPT and SEZ6L2*
non-protein coding lnc-MVP-3:20Possible ORF	*1062 nucleotides*	*29,863,852–29,868,050*	*Between CDIPT and SEZ6L2*
non-protein coding lnc-MVP-3:6Possible ORF	*799 nucleotides*	*29,863,852–29,868,050*	*Between CDIPT and SEZ6L2*
**KCTD13 - divergent transcript**	*1762 nucleotides*	*29,926,223–29,931,080*	*Between KCTD13 and Tmem219*
**YPEL 3 - divergent transcript**	*9026 nucleotides*	*30,096,430–30,105,456*	*Between YPEL3 and GDPD3*
**Coro1A antisense RNA 1**	*1395 nucleotides*	*30,183,393–30, 184,788*	*Between MAPK3 and Coro1A*

### MAPK3, MVP, Sez6l2, TAOK2, KCDT13 all potentially modulate cell signalling in 16p11.2 deletion models

**FIGURE 2:**
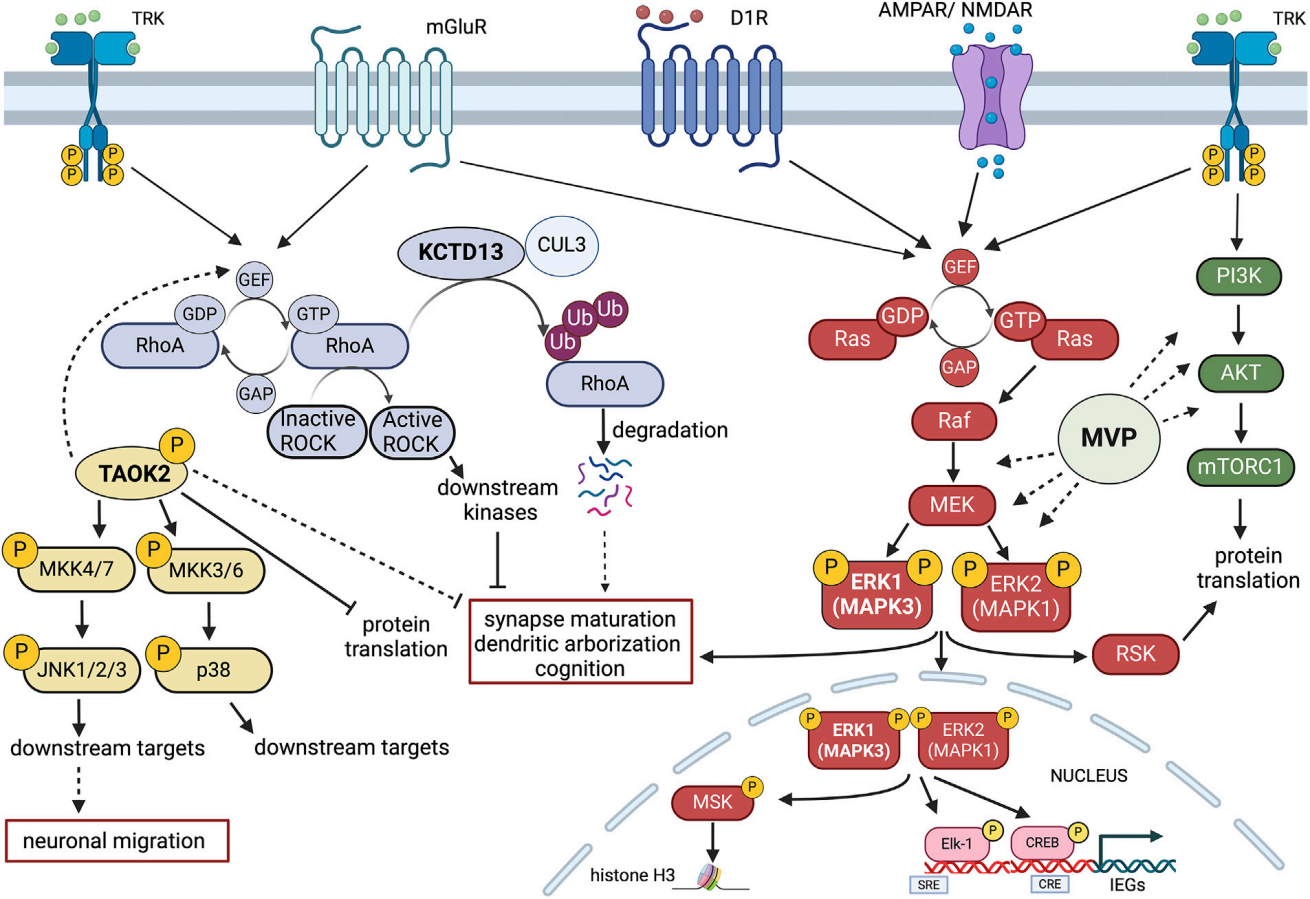
Schematic representation of potential signalling Interactions among four key 16p11.2 CNV genes. MAPK3 (ERK1), TAOK2, KCDT13 and MVP may interact at the intracellular level in modulating neuroanatomical, synaptic and cognitive functions. Gene dosage alterations present in either 16p11.2 deletion or duplication syndromes could imbalance the coordinated cellular activities of these genes. Multiple receptor systems can activate the Ras-Raf-MEK-ERK1/2 signalling pathway that controls both gene expression and chromatin remodelling. ERK1 (MAPK3) and ERK2 (MAPK1) also interact with the PI3K-AKT-mTORC1 pathway in modulating protein translation. One important aspect of MAPK3/MAPK1 signalling modulation is that MAPK3 gene dosage may shift the balance between the two kinase activities, resulting in different signalling intensities with consequences at the physio-pathological level. Major Vault Protein (MVP) may act as a scaffold protein for both ERK1/2 and mTORC1 signalling, thereby providing additional modulatory control. TAOK2 kinase stimulates multiple cytosolic and nuclear targets, including JNK1/2 and p38 MAP kinases that may interact with the ERK1/ERK2 pathway and the RhoA-ROCK kinase cascade. The action of TAOK2 may inhibit synaptic maturation and protein translation, potentially in opposition to ERK1/2. KCDT13 may facilitate RhoA degradation via CULLIN 3 (CUL3) interactions, also potentially antagonising some of TAOK2 functions. Figure has been created using Biorender.com.

MAPK3 codes for the extracellular signal-regulated kinase 1 (ERK1), a p44 protein kinase acting as a major signal transduction component of the Ras-Raf-Mek-ERK cascades (Figure 2). Interestingly, the MAPK1 gene, coding for p42 ERK2 kinase, is found in the distal portion of the 22q11.2 CNV region, another common chromosomal rearrangement implicated in NDD (Vithayathil et al., 2018; More et al., 2020).

The Ras-ERK signalling cascade has been implicated in a variety of cellular processes, from cell proliferation and cell survival to synaptic and behavioural plasticity (Fasano and Brambilla, 2011). However, unravelling its wide roles in development and in the adult brain is beyond the scope of this review.

Recent evidence indicates that targeting this pathway via pharmacological intervention may be a way forward to treat at least certain symptoms associated to 16p11.2 deletion and duplication. In order to understand the rationale of the potential therapeutic approaches based on ERK signalling modulation, we need to refer to the competitive model of ERK1 and ERK2 interaction, developed over the years by our laboratory (Mazzucchelli et al., 2002; Vantaggiato et al., 2006; Indrigo et al., 2010; Indrigo et al., 2023). Based on this model, ERK1 and ERK2 MAP kinases do not signal with the same intensity and, most importantly, they do not translocate into the nucleus at the same rate (Marchi et al., 2008). In fact, as recently described (Indrigo et al., 2023), ERK1 delays the entry of ERK2 MAPK, the most abundant of the two kinases, by specifically binding to a class of importins, the α1/KPNA2 group. This interaction occurs via the unique N-terminal domain of ERK1 to KPNA2 since this binding can be prevented by the administration of a cell penetrating TAT peptide coupled with the same ERK1 N-term domain. Either the downregulation of ERK1 expression/activity, via gene knock-out, viral mediated silencing, or via *in vivo* administration of the RB5 peptide results in neuronal survival, cognitive enhancement and increased structural and synaptic plasticity. Those remarkable effects are the direct cause of a global enhancement of ERK signalling in the brain (Indrigo et al., 2023).

Gene dosage of ERK1/MAPK3 is therefore a crucial determinant of the overall ERK1/2 activity. Following this reasoning, we originally speculated that ERK1/2 activity would be increased in a hemideleted condition, such as the 16p11.2 DEL. Using mouse models of the DEL condition, this prediction has been confirmed (Pucilowska et al., 2015). Importantly, in the DEL model, changes in cortical development and behavioural impairments have been rescued by treatments during embryonic development with inhibitory peptides of the Ras-ERK cascade (Papale et al., 2016; Pucilowska et al., 2018). The pharmacological treatment during gestation not only fully rescue those functional alterations but also brings back ERK1/2 activity to normal levels, as expected (Pucilowska et al., 2018). Interestingly, a later treatment during adulthood only partially improves behavioural deficits in the DEL model, suggesting that an earlier intervention may be preferable to maximise the therapeutic outcome. This evidence indicates that a manipulation of the activity of a single gene within the 16p11.2 locus may be an effective way to treat the deletion syndrome. Ras-ERK inhibitors are among the best characterised drugs available and they have been already tested in clinical trials for cancer therapy and in experimental models, also to rescue aberrant ERK activity in NDDs such as RASopathies (Papale et al., 2017).

As a mirrored situation, the 16p11.2 duplication syndrome may be characterised by a globally reduced ERK1/2 activity, due to the presence of three copies of MAPK3/ERK1. Currently, there is no published evidence supporting this claim but preliminary evidence in our laboratory suggests that this may be the case.

Additional evidence of the importance of ERK signalling in the 16p11.2 deletion syndrome comes from the observations on striatal dependent reward learning, in which ERK1 MAPK phosphorylation appears to be aberrantly elevated during acquisition of operant behaviour in response to sucrose as natural reward. Importantly, this effect was seen exclusively in males, highlighting the effect in sex-specific effects in NDDs. However, no attempts have been made to rescue this change (Grissom et al., 2018). Interestingly, in a parallel study on DEL mice linking spatial transcriptomic data and brain structural changes (MRI scans), three genes loosely linked to MAPK signalling were upregulated in DEL male mice only: MVP; Sez6l2; and TAOK2 (Kumar et al., 2018). To further investigate the relevance of such male-specific transcriptional changes, the same research group generated a triple MVP/Sez6l2/TAOK2 hemizygous mutant, using CRISPR/Cas9 technology (Kim et al., 2024). Interestingly, the triple hemideleted mutant mouse line shows male-specific behavioural alterations, such as hyperlocomotion and reduced reward learning in a progressive (but not fixed) ratio paradigm, largely overlapping with the phenotypes observed in the 16p11.2 DEL mutants. The same triple mutants did show a less pronounced phenotype, with less hyperlocomotion and no reward learning phenotype, when backcrossed in a MAPK3 hemideleted background. This observation is important but is subjected to multiple interpretations, considering the prominent role of MAPK3 and ERK signaling in striatal-dependent operant conditioning and reward learning (Mazzucchelli et al., 2002; Ferguson et al., 2006; Engel et al., 2009; Fasano and Brambilla, 2011; Shiflett and Balleine, 2011; Indrigo et al., 2023). Although ERK1/MAPK3 KO male mice show an enhancement of ERK1/2 signalling and striatal-dependent learning, neither the hemideleted nor the full ERK1/MAPK3 KO mice have been formally tested in the same protocol used by Kim et al. (Kim et al., 2024). Therefore, it is possible that the MAPK3 partial ablation may counterbalance the effect of the triple MVP/Sez6l2/TAOK2 mutation, effectively “rescuing” their phenotype by interfering with cell signalling changes. On the other hand, we recently showed that ERK signalling potentiation via pharmacological manipulation only enhances reward-based learning in WT females but not males (Indrigo et al., 2023). Considering that the treatment mimics ERK1/MAPK3 mutation, ERK1 MAP kinase may indeed play a little role in this specific form of learning in males. As a final note on this aspect, it is important to stress that differences in MAPK3 levels may lead to distinct phenotypes that may be more associated to the 16p11.2 duplication condition, including the higher propensity of the DUP carriers to develop schizophrenia. Indeed, MAPK3 mRNA increase in recent TWAS studies has been recently correlated to higher risk of psychosis and its mRNA levels have been found elevated in the prefrontal cortex of schizophrenic patients (Gandal et al., 2018; Gusev et al., 2018; Hall et al., 2020).

As further evidence supporting the central role of Sez6l2, TAOK2 and MVP, all three proteins have been loosely connected to ERK signalling. Major Vault protein (MVP) was originally discovered as a main component of the vault organelle, a ribonucleoprotein complex found in most eukaryotes (Berger et al., 2009). MVP is highly abundant in the CNS and considerable evidence has linked its function to growth factor receptors responses, serving as a potential scaffold protein for ERK and PI3K/AKT/mTOR signalling pathways (Figure 2) (Kolli et al., 2004; Kim et al., 2006; Dong et al., 2021; Wakatsuki et al., 2021).

In a recent report, we investigated how MVP may interact with other 16p11.2 genes in the definition of neuroanatomical phenotypes (NAPs), that are major determinants of brain structural changes and neuronal morphology (Kretz et al., 2023). Interestingly we found that MVP is the top driver among a set of additional 12 genes within the 16p11.2 region (PPP4C, ZG16, TAOK2, SLX1B, MAZ, Fam57b, BOLA2, TBX6, QPRT, SPN, HIRIP3, and DOC2A) to modulate NAPs in males. Remarkably, neither MAPK3 nor KCTD13 were implicated in NAPs, despite previous work for KCTD13 in Zebrafish suggesting a different scenario (Golzio et al., 2012). However, MVP and MAPK3 did show interaction in modulating anxiety-like behaviour and drug induced epilepsy, suggesting that these two proteins may work together in some of the pathological behaviours observed in the DEL carriers (Kretz et al., 2023).

Concerning the TAOK2 gene, accumulating evidence indicates that the Thousand and one amino-acid kinase 2 (TAOK2) gene product plays a central role in neurodevelopment and more specifically in the 16p11.2 deletion syndrome. TAOK2 gene is listed as a category 2-risk gene (strong association) in the SFARI GENE Scoring list (https://gene.sfari.org/database/human-gene/TAOK2). Moreover, whole-genome and exome sequencing of ASD families identified 24 different variants in TAOK2 associated to autism. Importantly, TAOK2 KO mice and their hemideleted counterpart show dose-dependent cognitive deficits, anxiety and social behaviour impairments, as well as abnormalities in brain morphology, cortical development, connectivity, dendrite and synapse formation, all resulting from a reduced excitatory activity (Richter et al., 2019). These *in vivo* data are all consistent with previous *in vitro* observations. Further evidence suggests that TAOK2 action may require the downstream JNK1 and p38 MAPK signalling activation and induces PSD95 stability and dendritic spine maturation via Septin7 phosphorylation. (Moore et al., 2000; Zhou et al., 2004; Yasuda et al., 2007; de Anda et al., 2012; Ultanir et al., 2014; Yadav et al., 2017). Recent evidence demonstrated that TAOK2 acts as translational repressor by inhibiting the eukaryotic elongation factor eEF2 (Henis et al., 2024) (Figure 2).

Importantly, overexpression of mutated TAOK2α variants, but not the TAOK2β variants, impaired neuronal migration by destabilising microtubules and reduced JNK1 activation. This effect could be replicated in TAOK2 KO brains but also in the heterozygous 16p11.2 DEL animals, that displayed reduced levels of phosphorylated JNK1 and neuronal migration deficits, that could be partially rescued by ectopic expression of TAOK2α in in the developing cortex (Scharrenberg et al., 2022).

However, another signalling pathway is also central to TAOK2 function in spine formation and stability, at least in the cortex. In fact, RHOA GTPAse activity is significantly reduced in TAOK2 KO and HET mice. Importantly, incubation with RHOA activators increases spine formation in the TAOK2 KO preparations, suggesting a link between the two molecules and indicating that TAOK2 may exploit multiple signalling pathways (i.e., JNK1 and RHOA) to control distinct neurodevelopmental processes (Richter et al., 2019).

Albeit interesting, the potential role of RHOA signalling in promoting brain function is not entirely consistent with the observation that another 16p11.2 gene, KCTD13, may exert an opposite function. KCTD13 is a member of a superfamily of at least 20 genes forming a complex with CULLIN 3 (CUL3) ubiquitin ligase. These genes have been implicated in several neuropsychiatric conditions (Teng et al., 2019). Importantly, a major target of the KCTD13-CUL3 complex is indeed RHOA, that is degraded, and its downstream signalling is attenuated in WT condition (Figure 2) (Lin et al., 2015).

Accordingly, the KCTD13 KO mouse model shows a reduction of the number of functional synapses, with a decrease of dendritic length, complexity, and dendritic spine density due to increased levels of RHOA. These alterations may be reversed by RHOA inhibition rather than RHOA activation (Escamilla et al., 2017). Additional evidence supports the negative role of RHOA signalling in behavioural deficits in a different KCTD13 KO mouse model, displaying no major differences between homozygous and heterozygous deleted animals and showing strong similarities with the 16p11.2 DEL model (Arbogast et al., 2019). In this KCTD13 KO model, as well as in the 16p11.2 DEL model, learning and memory deficits could indeed be rescued by an inhibitor of the Rho-associated protein kinase ROCK (Martin Lorenzo et al., 2021).

It remains to be established whether these contrasting observations about RHOA signalling are real or due to the fact that this molecular pathway is differentially implicated in distinct aspects of the pathology. Certainly, these findings underscore the complexity of the mechanisms associated with the 16p11.2 deletion.

The last gene that has been shown to play a significant role in some of the 16p11.2 deletion phenotypes is Sez6l2. This is a transport receptor transmembrane protein, required by the aspartic protease Cathepsin D for its endosome/lysosome localisation. Inactivating mutations or mislocalization of cathepsin D lead to neuronal dysfunctions including microcephaly, seizures, and cognitive and psychomotor defects. SEZ6L2, also known as BSRP-A (Brain-Specific Receptor A), is predominantly expressed in the brain and has been associated to febrile seizures, bipolar disorder and autism, together with the other two members of the seizure-related gene six family, SEZ6, SEZ6L. Functional studies on SEZ6L2 have also implicated this protein in neurite outgrowth. In addition, the triple mutant of SEZ6, SEZ6L and SEZ6L2 showed cerebellar deficits and a potential link with PKCα signalling activation, thus providing a possible functional link with other genes in the 16p11.2 locus, such as MAPK3, MVP, KCDT13 and TAOK2 (Miyazaki et al., 2006; Kumar et al., 2009; Boonen et al., 2016).

### Genes within the 16p11.2 region with limited functional information available

#### SPN

The sialophorin, or CD43, is a glycoprotein expressed on the surface of T lymphocytes promoting adhesion and activation during immune responses. This gene, together with CORO1A and KIF22 have been suggested to play a role in reduced lymphocytes count in 16p11.2 DEL patient presenting a concomitant low dosage of BOLA2 duplicone, located on 16p11.2 BP4-BP5 flanking region (Giannuzzi et al., 2019; Giannuzzi et al., 2022). In male 16p11.2 DEL mice, SPN protein is overexpressed mainly in telencephalic and cerebellar regions presenting decreased fiber density (Kumar et al., 2018).

#### QPRT

The quinolinate phosphoribosyltransferase is a key enzyme involved in the catabolism of quinolinate, an intermediate in the synthesis of nicotinamide adenine dinucleotide (NAD). QPRT-mediated NAD biosynthesis plays a fundamental role in neuronal differentiation and neurite growth during development (Haslinger et al., 2018; Neves et al., 2022). QPRT expression is reduced in cell lines derived from 16p11.2 DEL patients, and its knock-down in neuroblastoma cells during differentiation significantly altered neuritic growth and complexity, whereas QPRT knock-out (KO) increased cell death during differentiation (Haslinger et al., 2018). Furthermore, QPRT KO leads to the downregulation of several genes, including GABRB3, SNTG2, KCNQ, CNTNAP2. These genes are implicated in the formation of GABAergic synapses formation and are associated with ASD and epilepsy (Haslinger et al., 2018). QPRT also interacts with neuroligin 3 (NLGN3), a postsynaptic transmembrane protein involved in synapse formation and neuron-glia connections which is often found mutated in ASD patients (Shen et al., 2015).

#### ZG16

Human zymogen granule protein 16 is highly expressed in mucus-secreting cells its overexpression significantly suppresses tumour growth through T cells-mediated immune response activation (Meng et al., 2022). Although its role in 16p11.2 CNVs is unclear, it has been correlated with increased size of several brain areas in mice selectively ablated for this single gene (Kretz et al., 2023).

#### KIF22

Kinesin family member 22 (KIF22) has been shown to mediate cell proliferation by regulating mitotic spindle, microtubules stability (Tokai et al., 1996; Tokai-Nishizumi et al., 2005), as well as the expression of CDC25C, a cell cycle regulator (Nilsson and Hoffmann, 2000).

Among all genes of 16p11.2 region, KIF22 is specifically enriched in neural progenitors in G2/M phase, while its expression levels significantly decline as cells become post-mitotic (Morson et al., 2021). During CNS development, KIF22 transcripts are clearly segregated in the ventricular and subventricular zones, where cortical progenitors arise. KIF22, together with ALDOA, HIRIP3, PAGR1, and MAZ are also expressed in the ventricular zone of the ganglionic eminences where interneuron progenitors reside, supporting the hypothesis that 16p11.2 CNVs may disrupt the excitatory and inhibitory components of the developing brain. KIF22 dosage seems to regulate neurogenesis by affecting the length of the cell cycle, thereby determining the proliferative or neurogenic fate of the progenitors. Kif22 gene dosage variations linked to 16p11.2 CNVs would thus affect cell-cycle kinetics and perturb neuronal formation during development (Morson et al., 2021). In Zebrafish, KIF22 and ALDOA are the only deletion “dosage sensor” genes for 16p11.2 CNVs, meaning that their functional levels are sensitive to hemizygosity and thus lead to a pathological phenotype. In particular, reduction of KIF22 is associated with abnormal brain morphology and bent tail, as well as deficient axonal development (Blaker-Lee et al., 2012). The same effect on axonal development was observed in *Drosophila*, in which silencing of KIF22 homologs leads to the development of ectopic innervation and off-target recurrent axon branches in the neuromuscular junction (Park et al., 2016). Interestingly, these results are in agreement with nerve regeneration studies conducted in rats, where Kif22 has been identified as a potential therapeutic target for promoting peripheral nerve injury repair via Schwann cell proliferation and migration (Lu et al., 2022).

#### MAZ

The MYC-associated zinc finger protein is a transcription factor involved in several, highly specific, molecular processes related to gene expression and cell cycle, leading to differentiation and developmental effects. During development, MAZ is a cofactor of CTCF during chromatin insulation of active and repressed genes within the Hox clusters (Ortabozkoyun et al., 2022), specifically involved in the differentiation of motor neurons in vertebrates. Among its targets, MAZ regulates the expression of several WNT morphogens, involved in the correct morphological development of different organs. For instance, MAZ has been related to birth defects in the genitourinary tract, that are commonly observed in the context of 16p11.2 CNVs (Haller et al., 2018) as well as in eye development, also presenting comorbidity in 16p11.2 DEL and DUP patients (Medina-Martinez et al., 2020). As KIF22 and ALDOA, MAZ is also enriched in the ventricular and subventricular zones compared to post-mitotic cells, suggesting a role in neurogenesis (Morson et al., 2021). As a transcription factor, MAZ takes part in the correct synchronization between neurogenesis and NOTCH1 mediated gliogenesis. It is well known that during development, NOTCH1 signalling is downregulated during neurogenesis and activated in gliogenesis, following a specific timing. The upregulation of ADAM10 activates NOTCH1-mediated gliogenesis. Liu and colleagues have demonstrated that MAZ enhances ADAM10 transcription in response to activation factors such as CT-1 in cultured neuronal progenitor cells (NPC) (Liu et al., 2016). In neuronal stem cells (NSC), MAZ regulates the expression of Rho-GDIγ, which in turn modulates the activity of Rho GTPases during neuritogenesis, axon formation and dendritic development in neuronal differentiation (Wang et al., 2013). Moreover, MAZ activity has been reported to enhance NMDA receptor subunit type 1 (NR1) promoter activity during neuronal differentiation of P19 cells, contributing to the assignment of the correct functional profile to differentiated excitatory neurons (Okamoto et al., 2002). Thus, changes in MAZ dosage related to 16p11.2 CNVs might be related to structural changes in neuronal/glial tissue balance.

#### PRTT2

Proline-rich transmembrane protein type 2 (PPRT2) is a membrane protein, located at synaptic contacts, playing a role in synapse formation during development. It is also a crucial component of the neurotransmitters’ release machinery, by interacting with SNARE proteins and synaptotagmins 1 and 2 (Valtorta et al., 2016). PRTT2 has been found enriched in Drd2+ MSNs in 16p11.2 deletion mice, together with KCTD13, Fam57b, Sez6l2 and CORO1A (Portmann et al., 2014). Mutations in PPRT2 have been associated with paroxysmal movement disorders (Heron and Dibbens, 2013), benign infantile familial seizures and autistic developmental regression (Zhang et al., 2024), as well ataxia and seizures in 16p11.2 DEL patients (Vlaskamp et al., 2019; Padmanabha et al., 2024). Importantly, correction of PRTT2 copy number in 16p11.2 DUP mice could rescue hypersynchronous activity and enhanced glutamate release in cortical circuits, seizure susceptibility and social deficits (Forrest et al., 2023).

#### PAGR1a

Pax-interacting protein 1 is a component of the histone methyltransferase MLL2/MLL3 complex, with possible role in DNA damage response (Cho et al., 2007; Gong et al., 2009). This protein, highly expressed in neural progenitors (Morson et al., 2021), appears to have a role in early embryonal development, as suggested by animal studies. For instance, loss of function of PAGR1a in Zebrafish is associated with reduced brain ventricle size and less defined midbrain-hindbrain boundaries (Blaker-Lee et al., 2012). In addition, null mice for PAGR1a are not viable, while the loss of one copy leads to abnormal development of extraembryonic tissues, such as the amnion, chorion, and visceral yolk sac (Kumar et al., 2014). A recent clinical report by Daum et al. identified three individuals, carrying a homozygous missense mutation in PAGR1a gene and showing microcephaly, severe developmental delay, dysmorphism, neurological deficits and death in infancy (Daum et al., 2022).

#### CDIPT

CDP-Diacylglycerol-Inositol 3-Phosphatidyltransferase (CDIPT) catalyses the biosynthesis of phosphatidylinositol and is highly expressed during foetal and neonatal brain development in rats (Nyquist and Helmkamp, 1989). CDIPT knock-down in *Drosophila* displays altered growth development of the neuromuscular junction, a model for studying synapse growth defects (Iyer et al., 2018). In addition, a missense mutation in CDIPT gene in Zebrafish causes photoreceptor cells death and cataract (Murphy et al., 2011).

#### DOC2A

Double C2-like domain-containing protein alpha (DOC2A) is a cytosolic protein interacting with SNARE complex and phospholipids, acting as a Ca2+ sensor and mainly expressed in glutamatergic neurons, triggering glutamate release (Courtney et al., 2018). In the context of neurodevelopmental disorder, it has been found specifically overexpressed in 16p11.2 DEL male mice in brain regions with decreased fiber density (Kumar et al., 2018). Interestingly, DOC2A-KO mice display abnormal morphology of hippocampal neurons in the dentate gyrus, defective hippocampal activity as well as social deficits and repetitive behaviours. DOC2A functions appear to be regulated by the Ca2+ binding protein Secretagogin (Wang et al., 2023).

#### ALDOA

Aldolase A (ALDOA) is a glycolytic enzyme which catalyses the conversion of fructose 1,6-biphosphate into glyceraldehyde 3-phosphate and dihydroxyacetone phosphate. ALDOA is highly expressed in neural progenitors and its expression declines as the cells become post-mitotic (Morson et al., 2021). It is also part of ERK1/2 interactome during epidermal and neuronal differentiation (von Kriegsheim et al., 2009). Knock-down studies in *Drosophila* demonstrated the involvement of this gene in the locomotor function, specifically in climbing activity. In addition, ALDOA knock-down produces altered cell counts and patterning of different cell types, including increased photoreceptors neurons and misplaced bristle cells (Iyer et al., 2018). In Zebrafish embryos, ALDOA loss of function is associated with no response to touch (Blaker-Lee et al., 2012).

#### PPP4C

Protein phosphatase 4, catalytic subunit (PPP4C) plays a role in several cellular processes, such as microtubule growth and organization, DNA damage checkpoint recovery, apoptosis, and TNF-alpha signalling (Chen et al., 2008). However, its role in the context of 16p11.2 syndromes has not been completely elucidated. It has been found overexpressed in 16p11.2 DEL mice specifically in regions with decreased fiber density (Kumar et al., 2018), while studies in Zebrafish suggest the role of PPP4C in locomotor function and axon growth (Blaker-Lee et al., 2012).

#### TBX6

T-box transcription factor 6 (TBX6) is a putative DNA-binding protein involved in the specification of paraxial mesoderm via SOX2 regulation (Takemoto et al., 2011). Heterozygous loss of function of TBX6 gene in human and mice has been associated with congenital anomalies of the kidney and urinary tract (CAKUT), thus relating this gene with the kidney defects often observed in 16p11.2 DEL carriers (Yang et al., 2020). In addition, the increased incidence of congenital vertebral malformations in 16p11.2 DUP patients could be ascribed to TBX6 dosage, as also suggested by mouse studies (Ren et al., 2020).

#### YPEL3

Yippee-like three is a member of putative Zinc-finger motif-coding genes and p53-regulated tumour suppressor (Kelley et al., 2010). Its role in 16p11.2 syndromes is unknown, although YPEL3 loss of function Zebrafish display abnormal brain morphology (Blaker-Lee et al., 2012).

#### GDPD3

Glycerophosphodiester phosphodiesterase domain-containing protein 3 (GDPD3) has lysophospholipase activity against several lisophospholipids (Ohshima et al., 2015). Loss of function Zebrafish for this gene is characterised by abnormalities in brain morphology (Blaker-Lee et al., 2012). To date, no other information is available regarding its specific role in neurodevelopmental disorders.

#### CORO1A

Coronin 1A (CORO1A) is an actin-binding protein previously associated with lymphocyte trafficking and homeostasis (Foger et al., 2006). Mutations in this protein have been found in patients with severe combined immunodeficiency (SCID) (Khoreva et al., 2024). Its function in the context of 16p11.2 CNVs has not been determined yet, although it is associated with low lymphocytes count in 16p11.2 DEL patients (Giannuzzi et al., 2022). Zebrafish carrying CORO1A loss of functions display abnormal body length, defective neural tubes, deficient axon tracts and movement defects (Blaker-Lee et al., 2012). In 16p11.2 DEL mice, CORO1A is enriched in Drd2+ MSNs (Portmann et al., 2014).

### Genes with unknown function

#### C16orf54

Chromosome 16 open reading frame 54 (C16orf54) is a protein-coding gene expressed in 11 normal tissues and mainly located in the cell membrane. Although its function is unknown, overall C16orf54 appears as a potential marker of the poor prognosis of various tumours, suggesting that C16orf54 may participate in the progression of tumours (Du et al., 2022). C16orf54 expression is significantly associated with various immune-related pathways, such as PI3K/Akt/mTOR and Wnt/Beta catenin signalling pathways, inflammatory response, and interferon-gamma response (Ding et al., 2023). However, its specific biological function is still obscure and its role in 16p11.2 CNVs has not been investigated yet.

#### C16orf92

Chromosome 16 open reading frame 92 (C16orf92) encodes for a testis-specific protein necessary for the fusion between the oocyte and the sperm (Fujihara et al., 2020). To date, no other information is available regarding additional biological functions or potential association of this protein with neurodevelopmental disorders.

#### Fam57b

Fam57b consists of three variants upregulated during adipogenesis although at different degrees/timing. Fam57b mediates the production of lactosylceramide upon binding with a circulating metabolite of vitamin D with possible role in bone fracture healing (Martineau et al., 2018). Ceramides are precursors of sphingolipids, and their dysregulation can affect vesicle fusions and endocytic recycling. Ceramide derivatives interact with SNARE docking complex and recruit Munc13, the binding partner of Doc2A (calcium sensitive exocytosis regulator) thus enhancing calcium intracellular flow (Camoletto et al., 2009). McCammon et al. identified a subset of interacting 16p11.2 pairs of genes affecting brain and ventricle morphology in Zebrafish. In particular, DOC2A and Fam57a interacts to affect propensity to seizures and body size (McCammon et al., 2017). Its loss of function causes movement defects in Zebrafish, no response to touch and axonal defects (Blaker-Lee et al., 2012). In addition, Fam57b knock-down in *Drosophila* causes altered growth development of the neuromuscular junction (Iyer et al., 2018). Fam57b has been found enriched in Drd2+ MSNs in 16p11.2 DEL mice (Portmann et al., 2014), however its specific function in the context of neurodevelopmental disorders is currently unknown.

#### TMEM219

TMEM219 encodes for a transmembrane protein also known as IGFBP-3R (Insulin-like growth factor binding protein-3 receptor). It interacts with IGFBP-3 to mediate caspase-8-mediated apoptosis and tumour suppression in prostate and breast cancer xenografts (Ingermann et al., 2010). TMEM219 interacts with chitinase 3-like-1 (Chi3l1) and the multimeric complex formed by TMEM219/Chi3l1/IL-13Rα2 activates MAPK, AKT and Wnt signalling, inhibits oxidant-induced apoptosis in the lung, induces TGF- β1 and promotes melanoma metastasis (Lee et al., 2016). However, its role in 16p11.2 CNVs is currently unknown.

#### HIRIP3

HIRIP3 is highly expressed in human and mice embryos, in both cortex excitatory and striatal medium spiny neuron progenitors, as well as in the adult human brain (Morson et al., 2021). Early evidence suggested that HIRIP3 may be part of a histone H3.3 chaperone complex along with HIRA (Magnaghi et al., 1998; Ricketts et al., 2019), while HIRIP3 seems to partner with histone H2A/H2B (Assrir et al., 2007). HIRIP3 loss of function is known to produce movement defects in Zebrafish (Blaker-Lee et al., 2012).

#### Ino80e

Ino80e is a component of Ino80, an ATP-dependent chromatin remodelling complex. Although the specific role of Ino80e subunit is unknown, studies in yeast support the role of Ino80 complex in chromatin remodelling, DNA replication, stabilization of replication fork and resumption of replication after stress (Papamichos-Chronakis et al., 2006; van Attikum et al., 2007; Papamichos-Chronakis et al., 2011). Loss of function of Ino80e in Zebrafish is associated with defective neural tubes and abnormal body length (Blaker-Lee et al., 2012). This gene has been also found overexpressed in regions with decreased fiber density in 16p11.2 DEL male mice (Kumar et al., 2018).

#### Asphd1

The role of aspartate beta-hydroxylase domain-containing protein (Asphd1) is still unknown. In general, aspartate beta hydroxylases are rarely expressed in normal adult tissues, while they are overexpressed in several malignancies thus mediating cell proliferation and metastasis. They also appear to be a downstream target of ERK/MAPK and PI3K pathways (Hou et al., 2018).

### Non-coding RNAs in the 16p11.2 region

Non-coding RNAs (ncRNAs) are a class of RNAs that do not encode for proteins and include ribosomal RNAs, transfer RNAs and regulatory non-coding RNAs, such as long non-coding RNAs, micro-RNAs, PIWI-interacting RNAs and circular RNAs.

Regulatory ncRNAs are classified by their size and subdivided in long nc-RNAs (>200 nucleotides) and short ncRNAs (<200 nucleotides). Regulatory ncRNAs can modulate the expression of homeotic genes and target chromatin remodelling complexes. In addition, they can regulate mRNA stability and protein translation (Amaral et al., 2008).

MicroRNAs, a class of small single-stranded RNAs (∼21 nucleotides), are particularly abundant in the brain. They can regulate gene expression via RNA-induced silencing complex (RISC)-mediated translational inhibition or, very rarely, via mRNA cleavage by binding to the 3′untranslated region (3′UTR) of target mRNAs (Vaishnavi et al., 2013).

ncRNAs have been involved in development and in differentiation (Amaral et al., 2008) and their altered expression has been linked with several neurodevelopmental disorders, such as ASD, Fragile X syndrome and intellectual disability (Zhang et al., 2019). Several miRNAs also play an important role in controlling gene expression programs during development by targeting genes like Notch, Nodal, and Hedgehog. In addition, miRNAs are known to be involved in brain development and dendritic spine morphology (Tekin et al., 2022). Interestingly, peripheral microRNAs could represent potential biomarkers for brain disorders due to their association with the neuroendocrine and neuroimmune system as well as potential therapeutic targets (Tekin et al., 2022).

By using RNAcentral (https://rnacentral.org), we identified ∼30 ncRNAs within the 16p11.2 region (see Table 3; Figure 1), including miRNAs, long ncRNAs and three are short ncRNAs, mostly with unknown functions.

Interestingly, has-miR-3680-3p has been recently found upregulated in the peripheral blood of bipolar disorders patients compared to controls. This miRNA targets MAOA gene, encoding for the enzyme responsible for the degradation of biogenic amines, and thus associated with mood disorders (Tekin et al., 2022). In addition, has-miR-3680-3p has been also involved in oesophageal squamous cell carcinoma and in osteoarthritis (OA) (Shi et al., 2019; Xie et al., 2022).

By investigating the presence of miRNAs in five groups of CNVs, Qiao et al., 2013 demonstrated an increased number of miRNAs in *de novo* CNVs compared to familial CNVs and common CNVs found in subjects with idiopathic or syndromic ID and neurotypical individuals. Although the number of miRNAs in familial CNVs is lower than in *de novo* CNVs, it is higher than in common CNVs. Within the 16p11.2 locus, they found two miRNAs (miR-3680-3p and miR-3680-5p) associated with the 16p11.2 paternal duplication, whereas the 16p11.2 *de novo* deletion had no miRNA content. The role of these miRNAs and their targets in CNVs has not been identified yet (Qiao et al., 2013).

## Potential therapeutic approaches beyond the 16p11.2 genes

The focus of this review is the role of the genes located within 16p11.2 CNV in the pathophysiology of these NDDs. However, accumulating evidence indicates that therapeutic approaches to treat either 16p11.2 deletion or duplication may be effective without directly targeting genes located within this CNV region. Most of those approaches are based on the correction of excitation/inhibition (E/I) imbalances, frequently observed in different animal models of NDDs (Lee et al., 2017).

The most promising approach for 16p11.2 deletion is based on the use of GABA-B receptor agonists, such as R-Baclofen (Arbaclofen). This is a safe off-patent drug, and its racemic version has already been approved by both the FDA (Food and Drug Administration) and the EMA (European Medicines Agency) for the treatment of spasticity in multiple sclerosis and cerebral palsy. It is also commonly prescribed to children and adolescents with cerebral palsy (Dario and Tomei, 2004).

Most notably, Arbaclofen has been proven effective in reversing symptoms of models of Fragile X and has already been tested in clinical trials, although without definitive evidence of clinical efficacy (Berry-Kravis et al., 2012; Henderson et al., 2012; Zeidler et al., 2018).

More recently, the same drug has been tested in three different models of 16p11.2 deletion and the data generally support its efficacy in reverting some, but not all, behavioural symptoms (Stoppel et al., 2018; Gundersen et al., 2023).

This has led SFARI to initiate a recruitment for a clinical trial on 16p11.2 deletion carriers (https://clinicaltrials.gov/study/NCT04271332). Despite the obvious excitement about the first potential effective treatment for DEL carriers, it is important to highlight that very little is known about the mechanisms allowing Arbaclofen to change E/I in 16p11.2 deletion syndrome, a state that is apparently shared with Fragile X Syndrome.

Additional proposed treatments, already tested for Fragile X Syndrome, are based on the inhibition of metabotropic mGLUR5 receptors by using negative allosteric modulators (NAMs), such as mavoglurant and basimglurant (see for reference (Stoppel et al., 2017)). This observation is interesting and points to a potential dysregulation of protein synthesis downstream mGLUR5 receptors. However, it may need to be entirely reconciled with the additional evidence that glutamatergic function seems to be disrupted in a mouse model of 16p11.2 deletion and that chemogenetic activation of the prefrontal cortex pyramidal neurons may ameliorate cognitive symptoms (Tian et al., 2015; Berry-Kravis et al., 2017; Wang et al., 2018).

A final potentially relevant observation is that serotoninergic signalling via 5-HT1B receptors in the nucleus accumbens is disrupted in 16p11.2 deletion mice as well as in other ASD models. Both optogenetic or pharmacological stimulation of the serotonin pathway can reverse social deficits (Walsh et al., 2018; Walsh et al., 2021).

All the above approaches have been devised and tested to treat the 16p11.2 deletion syndrome. Translational research on the mirror condition, the 16p11.2 duplication syndrome, is lagging behind. However, in a recent paper, Npas4 has been identified as a potential therapeutic target. This GABAergic specific transcription factor has been found downregulated in the cortex of the DUP mouse model, in conjunction with an inhibitory transmission deficit and enhanced excitability. Remarkably, restoration of Npas4 expression in the PFC was able to rescue both synaptic and behavioural deficits in DUP mice (Rein et al., 2020). In addition, since Npas4 expression is negatively regulated by the epigenetic enzyme histone deacetylase 5 (HDAC5), administration of a HDAC5 inhibitor could restore GABAergic signalling and rescue behavioural deficits (Rein et al., 2022).

These studies are important, also because they suggest that pharmacological approaches aiming at ameliorating GABAergic activity, including the use of Arbaclofen, may be a potential way forward, as already evaluated for the 16p11.2 deletion syndrome. This evidence may also indicate that changes in E/I and circuitry disruption at the system level may converge onto the two conditions, despite the difference in gene dosage.

## Conclusion

16p11.2 deletion and duplications are amongst the most intensively studied neurodevelopmental syndromes. Over the last decade, considerable evidence has accumulated on these two genetically mirrored conditions, most notably in the domain of patient phenotyping and genotyping. Few hypotheses have been proposed to explain the reason why deletion and duplication carriers display opposite phenotypes (e.g., BMI, brain size), or at least different symptoms (e.g., schizophrenia and bipolar disorder are present only in duplication carriers). One possibility is that disease-specific symptoms may harbour from gene dosage differences within a subset of the 27 genes or non-coding RNAs located in the 16p11.2 CNV. However, no convincing experimental evidence in animal or cellular models is present to support this plausible, but difficult to test, hypothesis. Genetic approaches inducing mutations of the individual genes or pairs/trios of genes from a DEL to a DUP condition (and *vice versa*) could be employed to demonstrate that different behavioural or cellular phenotypes “appear” to be consistent with the mirror NDD. This would create “chimeric” models that are neither DEL nor DUP but do bear unique phenotypes of each state. We believe that the technology is now mature to perform this set of experiments, even in mammalian animal models, thanks to the advent of genome editing. However, the poor characterisation of the available deletion and duplication models, especially at the behavioural level, is still a major problem in the field. Before attempting complex but feasible genetic experiments, it will be imperative to characterise “at best” the available models, also considering the genetic background which is known to be remarkably important in modifying behaviour. In the context of 16p11.2 CNVs, see for instance (Arbogast et al., 2016) where deficits in social behaviour could be unmasked in a different genetic background. It is possible that, in the next years, more progress will be made using 2D and especially 3D cellular models generated from iPSCs, potentially “simplified” systems than *in vivo* models to study multiple gene function. However, even those approaches still have significant drawbacks (i.e., the availability of proper isogenic controls and the significant differences in differentiation protocols among different laboratories).

An even more complex problem is that the “core symptoms” of 16p11.2 deletion and duplication syndromes, such as ID and ASD, cannot be explained by differences in gene dosage. Maybe we have so far overestimated this aspect, considering that ID and ASD, or even epilepsy, arise from hundreds of different mutations, from “monogenic” forms (e.g., FXS or various RASopathies) to a variety of CNVs associated to NDDs. If developmental and behavioural biologists have learned any common lesson, it is clear that behavioural deficits may result from a large and potentially non-overlapping set of molecular and cellular process all impacting on the relevant circuitry. The prevalent idea that E/I imbalance may be at the core of any NDDs is important at the theoretical level but may not help much from the practical point of view. Very often in the past, translational approaches have been based on limited experimental evidence supporting E/I change, and clinical trials have been undertaken without substantial support drawn from work on patients. We desperately need more imaging and neurophysiological studies in patients, before considering applying drugs in a clinical setting. We believe that a better understanding of the patient’s endophenotypes and the identification of brain biomarkers for 16p11.2 deletion and duplication syndromes will better guide also translational research in experimental models. Thus, collaborative projects including cell, mice and patients’ studies will be the key to success in devising novel treatments.

One final note is about the syndromic nature of most NDDs, including 16p11.2 CNVs. The research is still too “neuro-centric” and most scientists, including us, have overlooked metabolic dysfunctions. These are instead likely to play a central role in syndromic NDDs, such as the 16p11.2 CNVs, and can clearly impact on the patients’ quality of life and their life expectancy. For instance, the 16p11.2 deletion syndrome is also associated with obesity and hyper insulinemic hypoglycaemia (Kostopoulou et al., 2019). A recent study highlighted unique and sex-specific metabolic signatures, also related to mitochondrial function, in three different mouse models of NDDs, including the 16p11.2 DEL (Menzies et al., 2021). It might be plausible that some genes in the CNV may be directly responsible for “peripheral” phenotypes, although this aspect has not been fully addressed in our review. However, we recognise that it is no longer sustainable to study neurodevelopmental disorders without considering the body as a whole. Investigating peripheral alterations in 16p11.2 CNVs, together with dissecting “brain-specific” phenotypes, will be crucial to inform and guide the development of future clinical interventions for these disabling conditions.
